# Aberrant newborn T cell and microbiota developmental trajectories predict respiratory compromise during infancy

**DOI:** 10.1016/j.isci.2022.104007

**Published:** 2022-03-01

**Authors:** Andrew McDavid, Nathan Laniewski, Alex Grier, Ann L. Gill, Haeja A. Kessler, Heidie Huyck, Elizabeth Carbonell, Jeanne Holden-Wiltse, Sanjukta Bandyopadhyay, Jennifer Carnahan, Andrew M. Dylag, David J. Topham, Ann R. Falsey, Mary T. Caserta, Gloria S. Pryhuber, Steven R. Gill, Kristin M. Scheible

**Affiliations:** 1 Department of Biostatistics and Computational Biology, University of Rochester, Rochester, NY, USA; 2 Department of Microbiology and Immunology, University of Rochester, Rochester, NY, USA; 3 Genomics Research Center, University of Rochester, Rochester, NY, USA; 4 Department of Pediatrics, University of Rochester, Rochester, NY, USA; 5 Department of Medicine, University of Rochester, Rochester, NY, USA

**Keywords:** Immunology, Microbiome

## Abstract

Neonatal immune-microbiota co-development is poorly understood, yet age-appropriate recognition of – and response to – pathogens and commensal microbiota is critical to health. In this longitudinal study of 148 preterm and 119 full-term infants from birth through one year of age, we found that postmenstrual age or weeks from conception is a central factor influencing T cell and mucosal microbiota development. Numerous features of the T cell and microbiota functional development remain unexplained; however, by either age metric and are instead shaped by discrete perinatal and postnatal events. Most strikingly, we establish that prenatal antibiotics or infection disrupt the normal T cell population developmental trajectory, influencing subsequent respiratory microbial colonization and predicting respiratory morbidity. In this way, early exposures predict the postnatal immune-microbiota axis trajectory, placing infants at later risk for respiratory morbidity in early childhood.

## Highlights

• T cells and microbiota in the first year of life advance in sync with infant age
• Perinatal events can disrupt T cell and microbiota developmental trajectories
• Atypical T cell and microbiota co-development predicts poor respiratory outcome

## Graphical abstract

**Figure:**
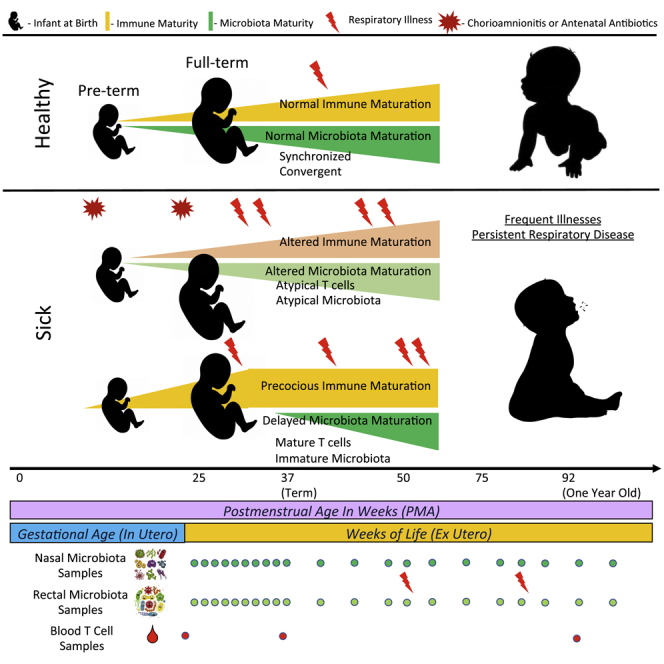

## Introduction

Function of the immune system and establishment of microbiota in human infants have profound impacts on subsequent health and disease. However, the factors influencing immune system and microbiota establishment and the extent of their interrelatedness are incompletely understood (Dimmitt et al., 2010; Rechavi et al., 2015; Lee et al., 2019). Normally, a developmental program determines major shifts in immune cell population maturation and distribution over the first 3 months of postnatal life (Olin et al., 2018). Though the exact stimuli for these shifts are unknown, it is increasingly clear that abnormal gut and respiratory microbiota during infancy associate with adverse outcomes such as atopy, stunted growth, and respiratory infection – outcomes that correspond to maladaptive immune system activity (Stewart et al., 2018; Grier et al., 2017; Bisgaard et al., 2011; Smith et al., 2013; Bosch et al., 2017). Two recent studies demonstrated that the nasopharyngeal microbiome and virome together predict infant respiratory tract infection, but these cross-sectional studies left unresolved the sequence of events preceding the observed relations of the microbiome and virome and their association to eventual adverse events (Korten et al., 2016; Man et al., 2019).

Reported adverse health effects following disrupted developmental processes support the concept of a critical neonatal window during which primary exposures and maladaptive immune responses risk lifelong health (Torow and Hornef, 2017). Recent reports also suggest that perinatal inflammation only transiently affects immune development (Kamdar et al., 2020). It is not known if such transient but early perturbations increase the risk of chronic disease specifically during particular developmental windows or operate more broadly. The strength of bidirectional influences between early microbiota and immune system development in humans is not well-understood, but such a concept is particularly relevant when considering the potential enduring impact on a long-lived adaptive immune system. Even less studied are the health effects of the microbiota-T cell axis in the first year of life, which is particularly concerning given the accelerated use of microbiome-targeting therapies designed to modulate immune-related outcomes in infancy (Reardon, 2014; Mcquade et al., 2019; Uchiyama et al., 2019; Watkins et al., 2017), motivating larger scale studies with sufficient longitudinal follow-up to identify immune-related outcomes. Here, we show, in a longitudinal birth cohort of infants born 23–41 weeks gestation, that microbial-T cell co-development advances in synchrony with the infant’s postmenstrual age, and deviation from typical or asynchrony between microbial-T cell trajectories increases the risk for poor respiratory outcome.

## Results

### Study design and demographics

**Figure 1:**
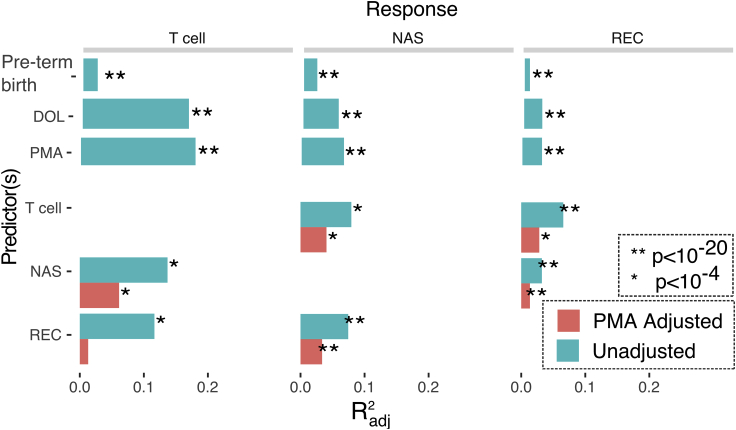
Systemic interactions between age, T cells, and microbiota The amount of variance in composition of nasal (NAS) and rectal (REC) microbiota and T cell immune populations that are explained by the predictors: preterm birth (gestational age at birth< 37 weeks), postnatal day of life (DOL), postmenstrual age (PMA), T cell population composition, and microbiota composition. Controlling for PMA, system interdependence was diminished but remained highly significant (red). All comparisons control for preterm birth. (∗p < 10^−4^, ∗∗p < 10^−20^, multivariate ANOVA).

Neonatal subjects (n = 267) born at 23–42 weeks gestational age (GA) were recruited within 7 days of birth at the University of Rochester from 2012–2016, as part of the NIAID-sponsored Prematurity, Respiratory, Immune Systems, and Microbiomes study (PRISM) (Figure 1B). In all, 122 preterm (PT, < 36 0/7 weeks gestation) and 80 full-term (FT, ≥ 37 0/7 weeks gestation) subjects completed the study to 12 months of age, corrected for premature birth, and were categorized as having or not having the primary outcome persistent respiratory disease (PRD) using previously published criteria (Pryhuber et al., 2015). Full term infants admitted to the NICU or requiring monitoring beyond normal newborn care were excluded from the study. Cohort demographics are shown in Table 1. Sufficient blood to perform T cell phenotyping by flow cytometry was collected at three predefined timepoints, from 55% of subjects at birth (cord blood), 61% of subjects at hospital discharge, and 38% at 12 months. For microbiota profiling, inpatient samples were obtained weekly and outpatient samples for PT and FT were obtained monthly, with additional sampling during acute respiratory illnesses. After sample processing, 16S rRNA gene sequencing, quality control, and removing subjects without immunophenotyping data, 149 subjects yielded 1748 usable nasal swab samples and 143 subjects yielded 1899 usable rectal swab samples. The median subject had 24 samples, with 28 days on average between samples. Finally, 109 and 117 subjects had sufficient combined T cell phenotyping and microbiota data to be included for immunome-nasal microbiota and immunome-rectal microbiota association analyses, respectively (Tables S1–S3).

**Table 1:** Subject demographics

	Preterm (n = 148)	Term (n = 119)
Gestational age (weeks)	29.8 ± 3.7	39.6 ± 1.0
Birthweight (g)	1406.6 ± 620.8	3471.5 ± 511.4
Female	71.0 (48.0%)	49.0 (41.2%)
Black or Asian race	46.0 (31.1%)	29.0 (24.4%)
Public Insurance	81.0 (54.7%)	59.0 (49.6%)
Maternal smoking postnatal	29.0 (19.6%)	15.0 (12.6%)
Delivered by cesarean section	94.0 (63.5%)	49.0 (41.2%)
Chorioamnionitis	7.0 (4.7%)	4.0 (3.4%)
Funisitis^a^	35.0 (23.6%)	0
Preeclampsia	26.0 (17.6%)	0
Antenatal steroids	121.0 (81.8%)	0
Postnatal steroids	47.0 (31.8%)	10.0 (8.4%)
Antibiotics while hospitalized, days (mean ± SD)	12.9 ± 16.5	0
Antibiotics after discharge, courses	1.1 ± 2.2	0.6 ± 1.1
BPD	25.0 (16.9%)	0
Supplemental O2 exposure by 14 days (Median FiO2 for first 14 days (IQR)	21.9% (21–27.8)	21% (room air only)
Postnatal infections (% with culture-positive bacteremia)	24.0 (16.2%)	0
Received breastmilk (any)	134.0 (90.5%)	92.0 (77.3%)
Months of >50% feedings by breastmilk	5.4 ± 4.5	3.0 ± 3.5
Number of illness visits/subject	1.2 ± 1.9	1.0 ± 1.8
Ventilator days	10.0 ± 18.3	0
PRD^b^	52.0 (35.1%)	17.0 (14.3%)

a Funisitis calculated on 144 PT and 27 FT subjects (placental pathology available).

b PRD measured in 122 PT and 80 FT

### Early T cell and microbiota co-development occur synchronously with age

We predicted, based on our and other previous studies, that T cell and microbiota evolution in the first year of life would proceed in an age-dependent manner. However, in a cohort of both full-term and preterm infants, age can be usefully defined in two ways: time since conception, which emphasizes the development process, or days since birth, which emphasizes external exposures. We previously examined several models relating microbiota-maturation and infant age (20) and found that in many microbial communities, postmenstrual age (PMA; defined as days since last known menstrual period, a proxy for time since conception) best indexes the progression of the communities. In other communities, days of life (DOL), i.e., postnatal exposures, essentially drive T cell and microbial maturation. Under the PMA-driven model, PT and FT subjects would exhibit distinct profiles at birth which would converge when PT infants achieved term equivalent PMA.

To understand the age-related factors influencing changes in the T cell immune compartment as well as the gut and respiratory microbiota longitudinally, we first applied unsupervised clustering approaches to reduce T cell populations and microbiota into biologically interpretable categories. The clustering algorithm FlowSOM identified 80 discrete populations of T cells using flow cytometry data (19): 50 from a T cell phenotyping panel (Tphe) and 30 from an intracellular cytokine panel (ICS). For the microbiota data, DADA2 was used to denoise and resolve the 16S rRNA amplicon sequence variants. We then compared the effect of preterm birth, PMA, and DOL on microbiota taxa and T cell subpopulations at a high level by using multivariate ANOVA to determine the explanatory power of each measure of age across all component microbes or T cell populations (Figure 1). Acknowledging that some covariates in Table 1 associated exclusively with PT birth, we first considered the effect of birth term alone. Preterm versus full-term status explained between 1% and 2% of the variance, and after adjusting for this factor, both DOL and PMA explained substantially more variance (3%–17%) in T cell, gut, and respiratory microbiota composition.

Anticipating that T cell populations and microbiota composition would show interrelated patterns of variation, we again applied multivariate ANOVA to quantify the amount of total variance the composition of one system could explain in another. All pairs of systems exhibited significant relationships with one another. The $R_{\text{adj}}^{2}$ ranged from 0.03 (nasal microbiota explaining gut microbiota) to 0.14 (nasal microbiota explaining T cells). However, given that all systems exhibited strong associations with age, we reasoned that much of the observed effects would be because of the common influence of age progression within subjects rather than direct action of one system on the other. Indeed, adjusting for PMA in these models attenuated the variance explained between systems by approximately 50%–90%, though all pairs were still significantly interrelated at p < 0.003. In all cases, PMA accounted for a greater proportion of variance when compared to DOL in the adjusted T cell-microbiota co-development models and achieved statistically superior BIC scores (Bayesian Information Criterion) (Table S4, p values in favor of PMA vs DOL ranging from 10^−50^ to 10^−153^).

Both age factors explain substantial amounts of variability, and indeed a model that includes both simultaneously leads to the lowest BIC scores of any model (Table S4). Given our primary interest in the independent role of host development across a wide range of gestational ages at birth, we focused on PMA as the univariate index of T cell and microbiota maturation. These results support PMA as an index of T cell and microbiota maturation, but further suggest a more complicated model in which these systems can co-occur independently of host age.

### T cell populations enriched in premature infants give way to a PMA-predicted trajectory that converges with full terms

**Figure 2:**
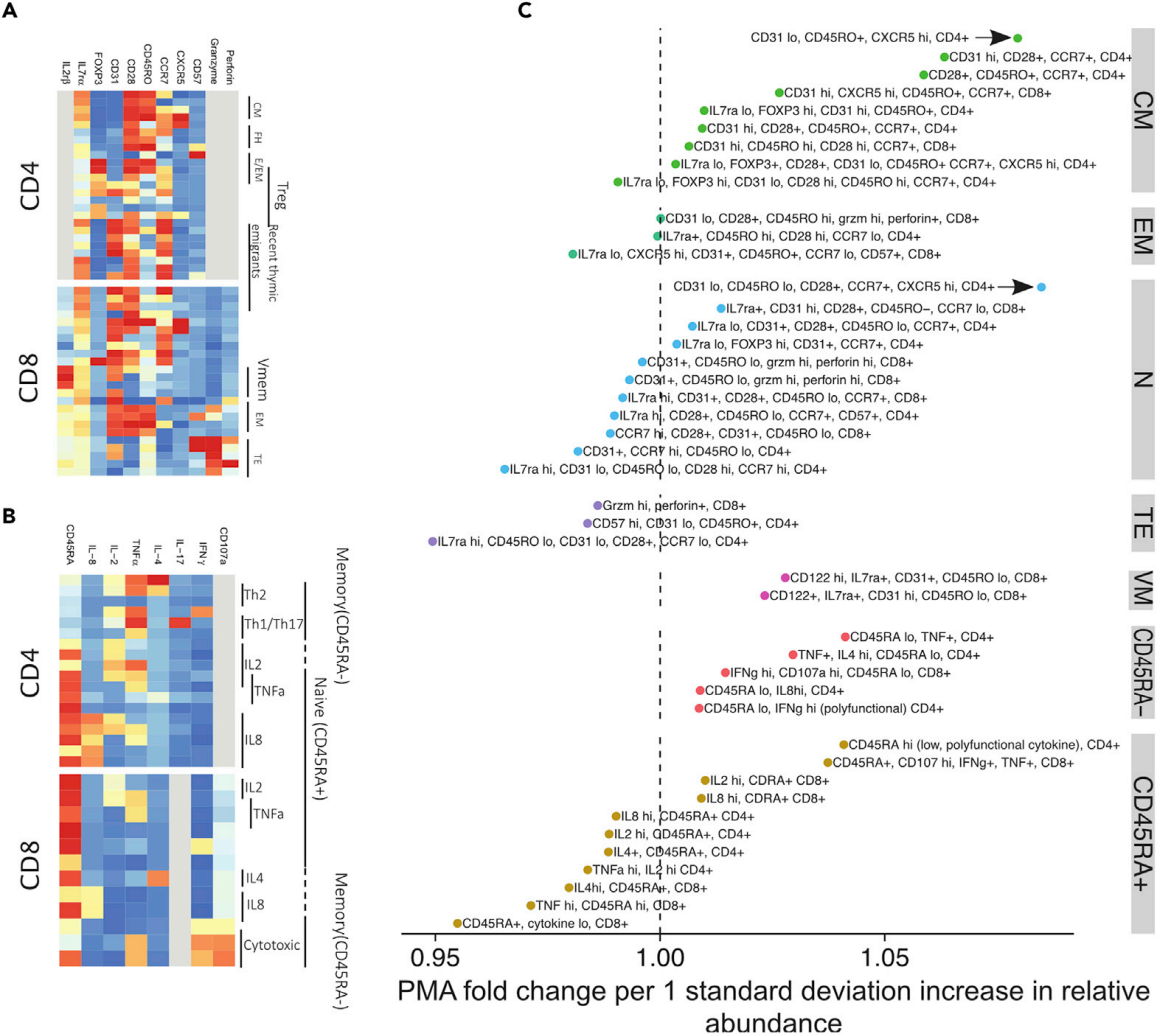
Early T cell development in preterm and full-term infants advances with postmenstrual age (A and B) T cells from flow cytometry performed on infants at birth, hospital discharge, and approximately one year of life were characterized by (A) phenotype (“Tphe”, unstimulated) and (B) cytokine function (“ICS”, stimulated *in vitro*) and clustered into subpopulations. The median fluorescence intensity of the flow parameters in the 79 clusters is shown. (C) T cell subpopulations from panels (B) and (C) that predicted sample PMA in a lasso regression are displayed, and their regression coefficients along the x axis. Populations that have inverse associations with PMA fall on the left of the dashed line, and vice versa. The x axis values indicate PMA fold-change per z-scored increase in the proportion of a subject’s cells assigned to that population. T cell phenotype subpopulations are grouped based on CCR7 and CD45RO expression (CM = central memory, EM = effector memory, N = naive, TE = terminal effector, and VM = virtual memory).

Using the FlowSOM-identified T cell populations in Tphe (Figure 2A) and ICS (Figure 2B), we examined age-related T cell population distribution changes in our cohort over time, from birth through one year. Using UMAP to visualize each sample’s T cell populations, we observed that full-term and preterm subjects clustered separately at birth, but converged by one year PMA (Figure S1), again supporting the notion of PMA as a dominant influence on this system. We log-transformed the PMA and applied elasticnet regression (see STAR Methods) to find the most important Tphe populations as statistical predictors of PMA. Log-transformation gave more sensitivity to the abrupt changes that occur early in life, and meant that the effect of the populations, as measured through their regression coefficients, can be interpreted multiplicatively as fold-changes. We then organized them into the following groups: effector memory (EM, CD45RO+, CCR7-, and CD28^−^), naive (N, CD45RO-, CCR7+, and CD28^+^), central memory (CM, CD45RO+, CCR7+), virtual memory (Vmem, CD8^+^, CD45ROlo, and CD122hi), and terminal effector (TE, CD45ROlo, CCR7-, and CD28^−^) (Bains et al., 2009; De Rosa et al., 2001; Gerlach et al., 2013; Buettner et al., 2015; Farber et al., 2014; Mahnke et al., 2013). ICS populations were first grouped into naive and memory (CD45RA+ and CD45RA-, respectively), and then named based on predominant cytokine profile.

T cells that displayed markers consistent with TE differentiation associated with the “youngest” PMA. Naive, then EM subsets, and finally CM emerged with advancing PMA. Naive populations showed considerable heterogeneity across PMA, indicating that variation in circulating T cells seen in early human development could potentially track to thymic or progenitor stages. CM populations that were present earlier typically carried a FOXP3+IL7rαlow T-reg cell phenotype (Figure 2A), supporting previous studies that indicate a predisposition to peripheral T regulatory cell (Treg) differentiation during human fetal development. Functionally, CD4^+^ T cells progressed from naive TNF-α and IL-2 high, then to IL-8 high, then to polarized (IFN-γ, IL-4, and IL-17), polyfunctional cells at later PMA. CD8^+^ T cells that were cytokine low or TNF-α positive were present at early PMA, then progressed through IL-4 and IL-8 positive, then cytotoxic CD45RA+. CD45RA low T cells were biased toward 12-month samples. Together, these results reveal a trajectory in which pauci-functional innate-like effectors and regulatory T cells are enriched at early gestational ages, giving way by term gestation to a more “typical” naive phenotype and a gradual gain of more polarized memory T cell phenotype postnatally.

### Atypical T cell phenotypes, but not cytokine function, are predicted by inflammatory exposures

**Figure 3:**
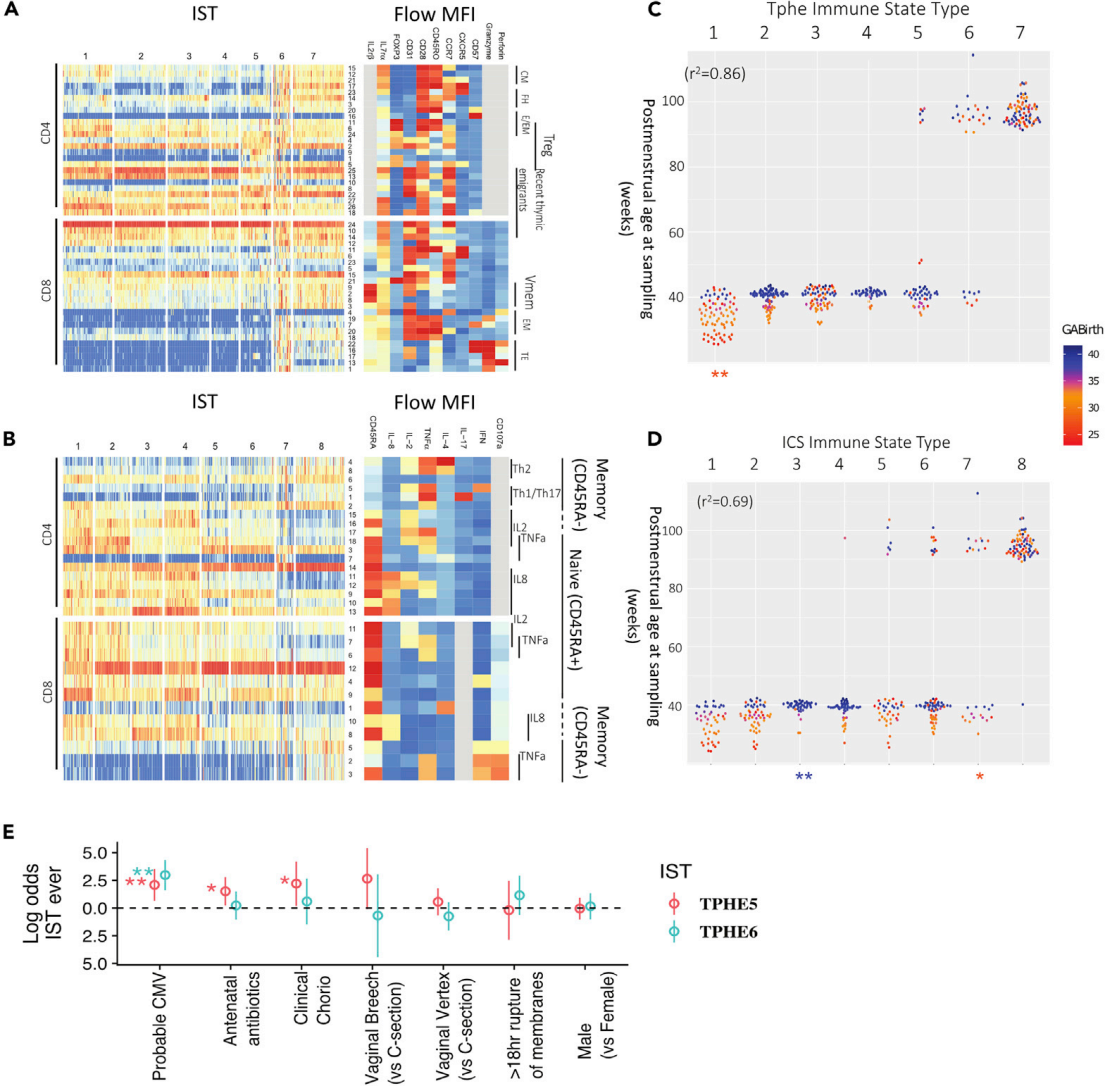
Immune State Types (ISTs) advance with postmenstrual age, and are perturbed by specific clinical exposures (A and B) T cell phenotype (A) and T cell function (B) immune state types (ISTs) were defined based on T cell population relative abundances. ISTs were enumerated according to the average postmenstrual age (PMA) at which they occur. Colors reflect relative abundances of component cell populations (rows); functional annotations and defining markers are shown in the heatmaps on the right. (C and D) The assigned IST vs PMA of sampling of (C) TPHE and (D) ICS. Each point represents a sample assigned to a given IST and is colored by gestational age at birth (GABirth) of the infant. The ANOVA coefficient of determination of PMA vs IST category is shown as the r^2^, whereas asterisks at the base of the dot plots indicate significant enrichment for either preterm (orange) or full-term (blue) samples within an IST, controlling for confounders and repeated measures (∗p < 0.05, ∗∗p < 0.01, ∗∗∗p < 0.001, two-tailed binomial test). (E) Joint logistic regression showing the log odds and 95% confidence interval of ever being in Tphe5 or Tphe6 given exposure indicated, controlling for gestational age (∗p < 0.05, ∗∗p < 0.01, ∗∗∗p < 0.001).

To characterize changes in the circulating T cell pool during infancy, we grouped Tphe and ICS samples into immune state types (ISTs) based on the relative abundances of their respective T cell populations using Dirichlet Multinomial Mixture (DMM) models (Figure 3). Each IST in this case represented an archetypal profile of T cell composition in terms of the relative abundance of the various T cell subpopulations, and samples were assigned to the IST which best explained their observed makeup. The seven T cell phenotype immune state types (Tphe ISTs, Figures 3A–3C) and eight ICS immune state types (ICS ISTs, Figures 3B–3D) were ordered according to their average PMA of occurrence. As a group, they explained a substantial amount of variance in PMA (ANOVA, *R*^*2*^ = 0.86 and 0.69, respectively, Figures 3C and 3D).

Once a normal progression was established, we next hypothesized that clinical exposures would predict deviation from the normal IST progression. To address this question, we used a joint logistic regression model that adjusted for GA, sex, race, mode of delivery, premature rupture of membranes, cytomegalovirus (CMV) infection, antibiotic exposure, and breastmilk to isolate the variables accounting for IST’s not following a PMA-determined progression pattern. Neither breastmilk intake nor postnatal antibiotic exposure predicted T cell state. However, several inflammatory or infectious conditions, including chorioamnionitis and exposure to antenatal antibiotics increased the odds of a subject ever entering Tphe5 by 9-fold (95% CI 1.2–66, p < 0.04) and 4.5-fold (95% CI 1.3–16, p < 0.02), respectively (Figure 3C). The increased abundance of CD57^+^ and cytotoxic CD8^+^ T cells in Tphe5 and Tphe6 raised our suspicion for prior cytomegalovirus (CMV) exposure, a strong stimulus for immunomaturation and known driver of high CD57 expression (Kern et al., 1999; Brenchley et al., 2003). It is important to note that no subjects in our cohort had stigmata or clinical evidence in support of a diagnosis of either congenital CMV infection or acquired CMV infection during hospitalization. CMV infection was detected by PCR on serial nasal swabs within 12 months in 18 subjects with T cell phenotyping. 40% of subjects entering Tphe6 (20-fold odds, p < 0.0001) and 19% of subjects ever entering Tphe5 (8-fold odds, p < 0.005) tested positive for CMV, compared to less than 8% of those subjects who were never in Tphe6 or Tphe5. Tphe5 early in life increased by 5-fold (p < 0.001) the odds of having Tphe6 at 12-months, but having Tphe6 early in life exhibited no association with Tphe5 status, indicating that Tphe5 may represent a less enduring immunophenotype whose potential is dependent on a window of immune development. Clinical exposures predicting T cell phenotype characteristics were not accompanied by changes in functional state types (ICS ISTs); in fact, ICS ISTs did not exhibit significant associations (FDR-adjusted p values > 0.70) with any variables considered, when controlling for age.

### Convergent microbiota community progression parallels T cell development in preterms and full-terms

Having characterized the compositional progression of T cell population profiles with respect to PMA, we performed a similar assessment of the microbiota to determine if a parallel pattern of maturation by PMA would hold. To summarize colonization of OTUs across samples, we used unweighted Unifrac distances between all samples within each body site to compute as a measure of β-diversity, which were then used to perform principal coordinate analysis (PCoA). For both body sites, the first principal coordinate (PC1) corresponded to PMA (Figure S3). Samples lower in PC1 tended to be taken before 40 weeks PMA, whereas advancement along the PC1 axis showed convergence of PT and FT subjects.

**Figure 4:**
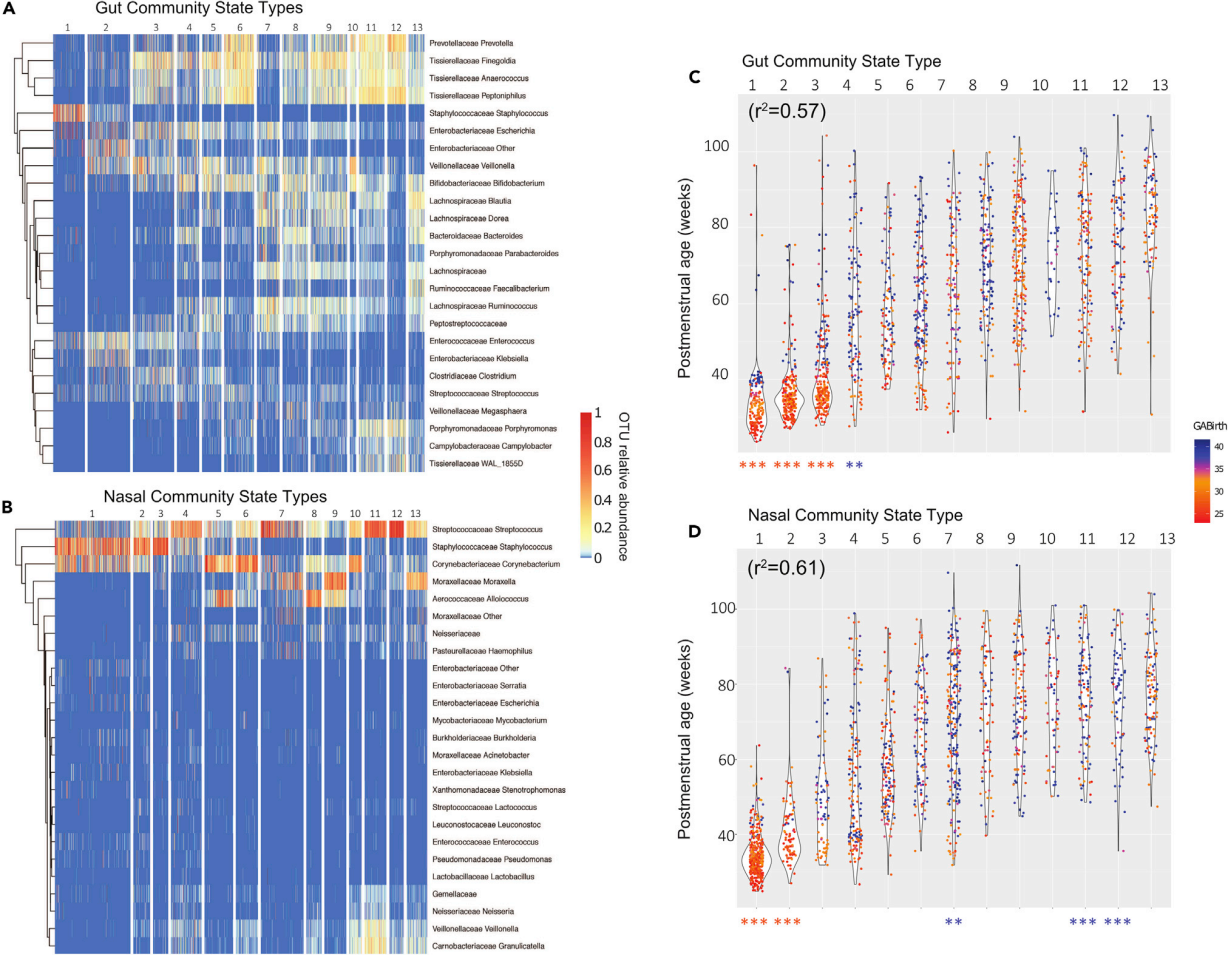
Premature birth influences long-term age-related respiratory and gut microbiota community progression Microbiota community profiling was performed on (A and C) rectal and (B and D) nasal samples obtained from 159 infants during regular surveillance and acute respiratory illness. (A and B) Microbiota community state types (CSTs) were defined for each body site based on sample composition, and the relative abundances of the top 25 most abundant genera were visualized using heatmaps, with samples as columns, clustered by CST. CSTs were numbered according to average PMA of occurrence. (C and D) Samples within each CST were plotted against subjects' PMA at the time of sample collection. Each dot represents a single sample, colored by the subject’s GA. r^2^ values show correlations between CST and PMA. Asterisks at the base of the dot plots indicate significant enrichment for either preterm (orange) or full-term samples (blue) within a CST controlling for confounders and repeated measures (∗p < 0.05, ∗∗p < 0.01, ∗∗∗p < 0.001, two-tailed binomial test).

We next applied a similar approach as performed on T cell populations using DMM modeling to partition samples into characteristic community state types (CSTs) based on their compositional profiles. Based on model fit and parsimony, 13 CSTs were defined for both respiratory (nCST) and gut microbiota (gCST) and were enumerated (1–13) according to the average PMA at which each CST occurred. CST 1 to 13 in the gut and the nose were strongly associated with PMA (ANOVA, *R*^*2*^ = 0.57 and 0.61, respectively) (Figures 4C, 4D, and S4). Both gCST1 and nCST1 were dominated by *Staphylococcus*. Gut and nasal CST’s later diverged when more niche-specific taxa took hold, including *Enterobacteriales* and *Clostridiales* in the gut and *Streptococcus* and *Corynebacterium* in the respiratory tract (Figures 4A and 4B). A small number of gCSTs and nCSTs occurring later in the first year of life, indicated by asterisks below Figures 4B–4D, were overrepresented by either PT or FT subjects after controlling for confounders as in the previous section and repeated measures using logistic mixed models. *Staphylococcus* and *Streptococcus*-dominant nCST1, nCST2, and gCST1 were more frequently found in PT subjects, as well as gCST2, gCST3. These findings independently predicted by gestational age at delivery, suggest infrequent but enduring microbial effects related to premature birth.

### Duration and timing differentially affect microbiota-T cell axis

The immune system and microbiota appeared to be regulated in tandem with advancing PMA in our cohort. We therefore hypothesized that microbiota and T cell associations that persist after adjusting for PMA, indicating asynchrony with infant development, might impact an infant’s health. To explore this hypothesis with respect to the longitudinal sampling scheme, which was relatively sparser for the T cell than for the microbiota, we considered if the number of days a subject spent in each CST, adjusting for the total length of surveillance, was associated with the T cell state at birth, discharge, and one year. A strength of this model is that it flexibly models temporal relations between T cells and the eventual or preceding microbiome state. Another strength is that it summarizes the microbiome and T cell state across time providing a single value for the subject on these repeated measures. We fit models on all pairwise combinations of CSTs and immunological parameters. Of the potential 6,318 possible associations between the 26 CSTs and 243 T cell parameters, we report only associations that are significant after adjusting for multiple comparisons.

The significant results of these tests, which corresponded to interactions between the T cells and microbiota present in our cohort, were visualized as networks (Figure S5). After multiple test correction, in a baseline model that only adjusts for premature birth, initially 166 pairs were found to be associated, which dropped to 49 pairs after adjusting for PMA, breastmilk exposure, inpatient antibiotic days, outpatient antibiotic courses, and mode of delivery, further supporting the overarching importance of PMA in our study.

**Figure 5:**
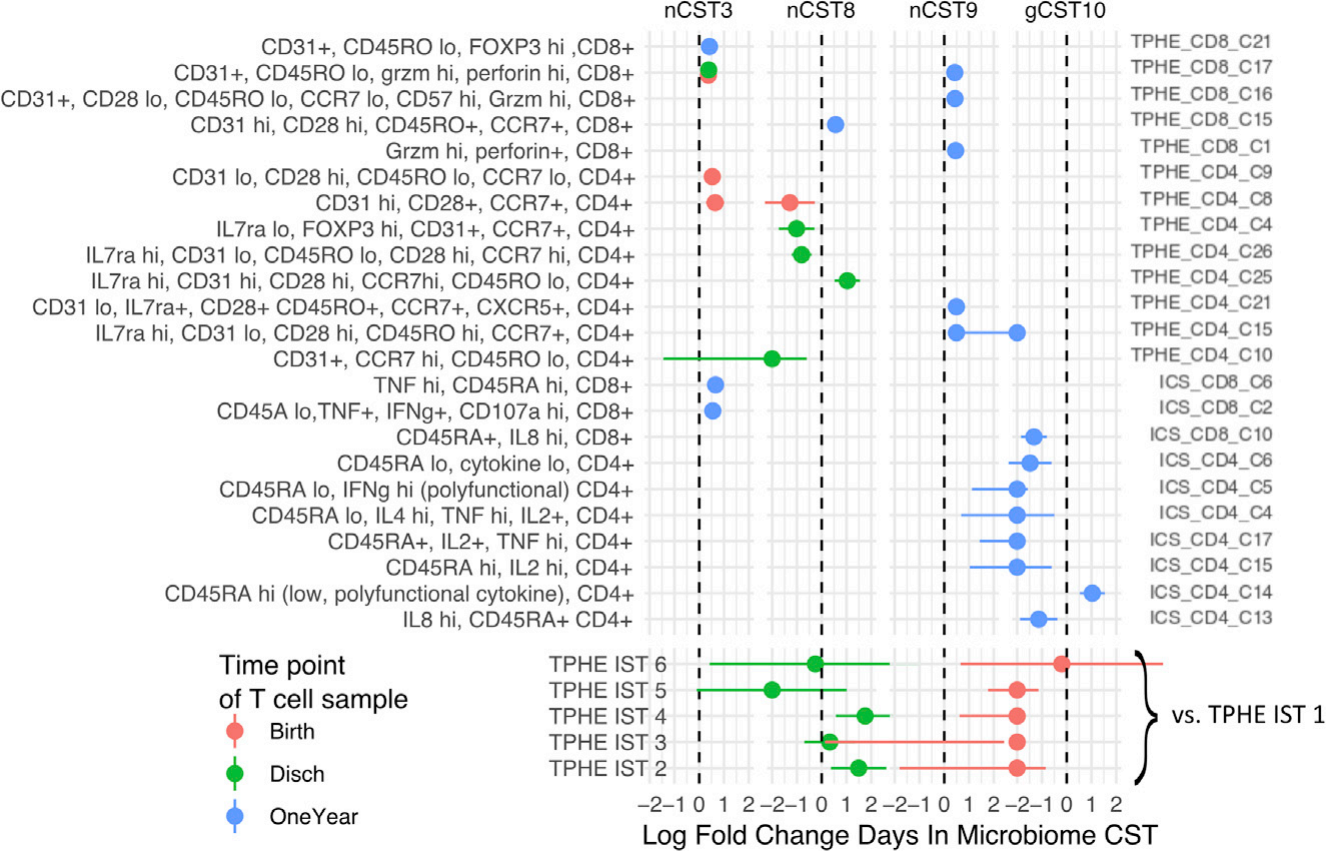
Bidirectional associations between the microbiome and T cell expansion Microbiota duration (days spent) in microbial community state types (CSTs) was modeled as a function of T cell features, controlling for gestational age at birth, mode of delivery, human milk consumption, and antibiotic exposure using quasi-poisson regression. The log rate ratio and its 95% confidence interval is shown for associations that were significant at 10% FDR.

In the 49 associations that persist in the adjusted model, four microbial states appear to be especially correlated with the immune state. The community types, nCST3, nCST8, nCST9, and gCST10 span over half of these significant associations. The relatively sparse associations that occur independent of PMA and often involve identical or closely-related T cell populations (Figures 5 and S5). Increased relative abundance at birth in two naive and early activated CD4^+^ populations, one CD31^+^ and one CD31^−^, tended to increase time spent in nCST3, which is a pauci-diverse CST dominated by Staphylococcus and Streptococcus, and found in both PT and FT from birth until roughly 60 weeks PMA. Increased relative abundance of several CD8^+^, granzyme, and perforin high effector-rich subpopulations at birth, discharge, and one year were also associated with nCST3. Lastly, at one year, subjects who spent longer in nCST3 tended to have higher levels of two cytokine-producing (IFNg+/TNFa+) and cytotoxic (107a+) CD8^+^ T cell populations.

Increased relative abundance at birth and discharge of Tphe5 and its individual clusters characterized by CCR7 high, early activated, and FOXP3+ regulatory CD4^+^ populations associated with shorter durations of nCST8. This CST, distinguished by its *Alloiococcus* dominance, was found at 40 to 80 weeks PMA. In contrast, TPHE IST 2 and 4, characterized by their enrichment for naive and RTE CD4^+^ and CD8^+^ T cells, predicted longer duration in nCST8, indicating that this may represent a more typical microbial-immune relationship. The nCST9, distinct in its predominant *Moraxella* species, was associated with increases in numerous cytotoxic and phenotypically memory CD4 and CD8 populations at one year. Lastly, longer duration of gCST10, enriched for the beneficial *Veillonella* and *Bifidobacterium* species, predicted lower frequencies of cytokine-positive CD4^+^ and CD8^+^ T cells at one year, but was strongly and positively associated with CD4^+^ T cells that lacked appreciable cytokine production upon stimulation, suggesting maintenance of a regulatory milieu.

Together these models suggest that there is an age-independent microbiota-T cell axis, and the bidirectional relationship between T cell phenotype and microbiota displays early T cell phenotypic differentiation predicting later microbiota, and T cell functional maturation following microbiota exposures.

### Sequence and timing of T cell-microbiota associated events predict infant respiratory outcomes

**Figure 6:**
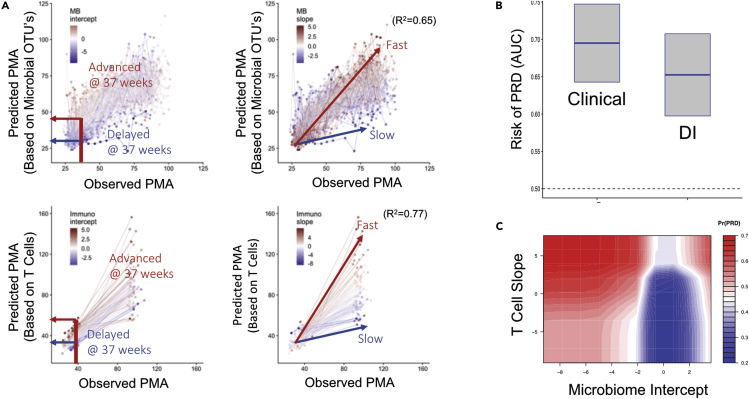
Mistimed Immune and Microbial Development Predict Respiratory Outcome Elastic net regression predicts a postmenstrual age (pPMA) based on both T cell populations and microbial operational taxonomic units (OTUs), separately. (A) The pPMA of a subject is plotted against the observed age (oPMA) at the time of sampling to establish an intercept at 37 weeks (left) and a slope (right), corresponding to maturity at term equivalent and rate of maturation over the first year, for both T cell and microbiota. Z-scores for each subject’s slope and intercept are indicated as color overlays (red - relatively advanced maturation at term equivalent/faster development, and blue - relatively delayed maturation at term equivalent/slower development). A “Developmental Index” (DI) was constructed using these four parameters: the z-scores of T cell and microbiota intercepts and slopes. (B) A random forest machine learning algorithm predicts persistent respiratory disease (PRD) from known risk factor clinical variables and from the T cell and microbiota-based DI. Boxplots show the area under the curve calculated for each set of variables (mean and standard error of the mean). (C) The contour graph demonstrates the two DI components, the microbiota intercept and T cell slope, with the best predictive strength for PRD risk, controlling for clinical factors. Blue = lower PRD risk, red = higher risk.

Observing sparse age-independent T cell-microbiota interactions, we hypothesized that maintenance of a typical longitudinal microbiome-immune axis is a marker of health, and the trajectories that deviated with respect to an infant’s PMA would predict respiratory morbidity in the form of PRD. One early indicator in support of this hypothesis was that *Alloiococcus* (dominant in nCST8 and negatively associated with Tphe5), for every 10% increase in relative abundance, there was a 1.4-fold reduction in the odds of a sample being taken during acute illness compared to healthy surveillance (1.1 to 1.8-fold, 95% CI, p value < 0.003), controlling for multiple confounders. However, to formally test our hypothesis that microbiota and T cells work together as age-dependent *systems* in maintaining respiratory health, we developed a quantitative model of the “normal” relationship between PMA and T cell and microbiota developmental trajectories, which was then used to assign a *developmental index* (DI) for each subject. For DI, we trained two sparse regression models that used the T cell populations and microbiota abundance vectors to predict log2-transformed PMA at sample collection. For each subject, two trajectory components were defined: first, the fitted intercepts of these models represented the predicted PMA at 37 weeks actual PMA, indicate the subject’s microbiota and T cell maturity relative to “normal” at 37 weeks PMA (see “immunological and microbial developmental indices” Methods for details), and second, the fitted slopes of the models indicate a subject’s *rate* of microbiota and T cell maturation over the first year, again relative to normal. Holding out a subject’s longitudinal record, the cross-validated models strongly predicted PMA using either T cell populations (R^2^ = 0.77) or bacterial taxa (R^2^ = 0.65) (Figure 6A). The four fitted DI parameter z-scores together (intercept and slope for microbiota and T cells) were then used to quantify mistiming with respect to age, or asynchrony between age, T cell and microbiota development, and its contribution to PRD.

We used random forest classification models to compare the predictive power of the four DI features alone to that of a set of known clinical risk factors for PRD. The clinical features were race, maternal education, sex, GA, birthweight, season at birth, and oxygen supplementation integrated over the first 14 days of life (Keller et al., 2017). In cross-validation, the clinical features predicted PRD with area under the curve (AUC) of 0.69 (0.59–0.79 95% CI), the strongest features being increased oxygen exposure, lower birthweight, and younger GA (Figure S6). When compared to clinical predictors, the developmental index had statistically equivalent skill in predicting PRD (Figure 6B, AUC 0.64, 0.54%–0.74 95% CI). Combining the clinical features and the developmental variables did not improve the AUC of the predictive model; however, indicating potential overlapping and equal effects between the two. Of the four components generating the DI, the microbiome intercept and immune slope had the largest variable importance scores. In exploring the functional relationship between PRD and these factors, we observed that an immature microbiota at term equivalent PMA increased the risk of PRD by over 2-fold, and this effect was magnified in subjects with accelerated T cell maturation (Figure 6C). These results indicate that microbial-immune axis trajectories are equally informative to known clinical risk factors when predicting outcomes in infants. Furthermore, the timing of T cell-microbiota co-maturation *relative to an infant’s age* appears to play a role in determining respiratory health.

## Discussion

Birth initiates a dynamic interplay between developmental programming, colonization and assembly of the microbiome, and differentiation and maturation of the adaptive immune system. In healthy infants, this process balances the accommodation of commensal microbiota, appropriate immune response to pathogens, and functional maturation of the organs at the mucosal interface between human host and environment. Here, we present results from a cohort of preterm and full-term human infants, in which respiratory microbiota, gut microbiota, and T cell systems were measured longitudinally with respect to several age measures and assessed for their combined strength in predicting respiratory outcomes. By creating new longitudinal models of microbiota composition and T cells, we were able to describe the co-development of these systems. We found T cells and microbiota exhibit structured patterns of progression, driven by age with pronounced differences between PT and FT infants in very early life, and indeed both days of life and PMA are important factors. In many cases, there was a tendency toward convergence postnatally, hence we focus on PMA as a synchronizing factor. Furthermore, outside of PMA-driven development, interactions occur between T cell population profiles and microbiota community structure, with early atypical or asynchronous immune and microbiota features initiating a cascade of events that predict respiratory disease in infants.

Our central observation of a patterned progression of the immune system and microbiota builds on previous studies demonstrating age-dependent shifts in gut microbiota and higher order innate and adaptive immune cell populations (Peterson et al., 2021; Olin et al., 2018; Roswall et al., 2021). Our study adds to these reports in several ways. We focused on deep T cell phenotyping, which was important because mouse models and human vaccine studies indicate that infant T cells, even those derived during gestation, may survive into adulthood and therefore may maintain an imprint of early exposures (Smith et al., 2018; Tough and Sprent, 1995). We found pauci-functional effectors and regulatory T cells to be enriched at early gestational ages, giving way by term gestation to a more “typical” naive phenotype and a gradual gain of more polarized memory T cell phenotype postnatally. The Treg bias is consistent with studies showing a predisposition to CD4^+^ Treg and Treg differentiation in human fetal-derived T cell progenitors (Hayakawa et al., 2017; Mold et al., 2010). Another parallel reported finding is that neonatal mice exposed to HSV-1 antigen have enhanced CD8^+^ T cell short-lived effector differentiation in human fetal-derived T cell progenitors (Smith et al., 2014, 2018; Rudd et al., 2013). Moreover, these differential T cell responses observed in mice persist and shape the immune response towards SLECs upon secondary challenge. Whether or not antigen-specific T cells, such as vaccine-induced, are differentially recalled later in childhood in those infants primed at a time when they harbored a Treg/effector phenotype seen at lower gestational ages has not been directly studied, but the PT or FT-biased IST’s at later PMA suggests that early perturbations may have sustained effects on the immune system.

To test these sustained effects, we applied a longitudinal approach, which, when ordered by average PMA of the sample, revealed a linear progression of immune and microbiota state types by PMA from birth to one year. A similar patterned progression was seen in both gut and nasal microbiota. Individual longitudinal trajectories that deviated from the average trajectory, for example, subjects who entered early into the Treg-enriched state type, Tphe5, were more likely to have been exposed to immune-modulating and microbiota-modulating stimuli (chorioamnionitis, antenatal antibiotics), and are also more likely to be colonized later with CSTs in an age-independent fashion. This pattern has also been shown in nonhuman primates, in whom intrauterine inoculation with LPS or infection causes expansion of dysregulated FOXP3+ CD4^+^ T cells (Rueda et al., 2016), as well as human neonates in whom chorioamnionitis or funisitis show an altered placental microbiome as well as sustained inflammatory transcription factor profile, including defects in FOXP3+ CD4^+^ T cell function (Rueda et al., 2015; Misra et al., 2015). By applying IST grouping, we were able to show that Tregs and effectors are expanded within the same Tphe5 IST, suggesting a state of dysregulation rather than successful immune suppression. In support of this interpretation, the CCR7-FOXP3+ CD4^+^ subpopulation associated with Tphe5 arises in inflammatory states and can contribute to immune dysregulation in CCR7 null mice (Smigiel et al., 2014). We did not directly test *in vitro* function of chorioamnionitis-exposed or antibiotic-exposed Tregs with respect to colonizing bacteria, but it is plausible that in newborns, early exposures shape both immediate immune system development and alter their responses to infection and niche receptiveness to an age-appropriate microbiota.

A growing set of birth cohorts with longitudinal observations suggest that immune system development and microbiome as independent variables predict health outcomes in infants. For example, previous reports and our analysis indicate that in children, *Alloiococcus* in the respiratory tract has protective associations against acute respiratory infections (Teo et al., 2015; Ta et al., 2018). In our cohort, *Alloiococcus*-dominant nCST8 was found more often in asymptomatic subjects, but was less frequent in infants with an early atypical immunophenotype. We tested the role of an infant’s age during a microbial-immune axis shift, and if these systems worked together to impact respiratory outcomes. In support of our hypothesis, we observed an age-specific microbiota-immune axis combined trajectory, and when altered, this axis predicted respiratory morbidity. Specifically, accelerated T cell trajectory appeared harmful, but this effect was attenuated by an advanced microbiome. Another recent study consistent with our finding showed that, in preterm infants, white matter injury of the brain was associated with early changes in T cells, stool microbiota and short-chain fatty acids, and these variables acted together to converge on an unfavorable outcome (Seki et al., 2021). Given that deviation from typical ISTs and CSTs was best predicted by antenatal infection or antibiotics, the pathologic cascade that links microbiome-immune axis with health outcomes is likely to be initiated before birth. The bidirectional effects are also evident in the continued effect of gut and nasal microbiota on T cell phenotype and function at one year. These windows of opportunity represent a challenging but promising target in preventing the leading cause of pediatric outpatient visits and hospitalizations, namely respiratory infections and exacerbations (Busse et al., 2010; Tregoning and Schwarze, 2010; Chen et al., 2019). However, our results also underscore the need to assess microbiota and immune systems within the context of one another and the infant’s physiologic development before considering interventions that could potentially disrupt a well-orchestrated, age-appropriate immune-microbial axis.

### Limitations of the study

As an observational human study, there are several limitations that need to be acknowledged. First, this study cannot confirm causal mechanisms between the microbiome, the immune state, and the disease, without making strong and fundamentally unverifiable assumptions about the type and nature of confounding; although we have attempted to adjust for known potential confounders of immune and microbiome development, undoubtedly others will be revealed in future studies that will need to be addressed. Dense longitudinal sampling of the microbiome in both healthy and ill states, however, offers some ability to quantify the temporal relationships between these two systems. This study used PMA as a univariate index to organize temporal relationships we observed. However, both day-of-life and PMA are important factors. Future work could consider more complicated multivariate or nonlinear relationships between PMA, day of life, microbiome, and immune states. Unavoidable limitations in infant blood sampling resulted in asymmetric sampling of microbiome and immune variables, but our deep and broad T cell profiling will allow future studies to sample more frequently with less volume focusing on the most relevant immune populations. Future studies will also benefit from metagenome and metabolomics to better resolve microbiota species and functional potential, as well as single-cell genomics to understand the molecular underpinnings of early T cell-microbiota co-development.

## STAR★Methods

### Key resources table

**Table:** 

REAGENT or RESOURCE	SOURCE	IDENTIFIER
**Antibodies**		

CD122 (Mik-β)	BD Biosciences	Cat#564688; RRID:AB_2738895
Perforin (dG9)	Biolegend	Cat#308114; RRID:AB_2284280
Granzyme B (GB11)	BD Biosciences	Cat#563389; RRID:AB_2738175
CD3 (UCHT1)	Biolegend	Cat#300436; RRID:AB_2562124
CD31 (WM59)	BD Biosciences	Cat#562855; RRID:AB_2737841
CD127 (HIL-7R-M21)	BD Biosciences	Cat#563225; RRID:AB_2738081
CD45RO (UCHL1)	BD Biosciences	Cat#563722; RRID:AB_2744413
CD8a (RPA-T8)	Biolegend	Cat#301045; RRID:AB_11219195
KLRG1 (13F12F2)	eBioscience	Cat#17-9488-42; RRID:AB_2573303
CD185 (RF8B2)	BD Biosciences	Cat#565191; RRID:AB_2739103
CD197 (G043H7)	Biolegend	Cat#353212; RRID:AB_10916390
Foxp3 (236A/E7)	eBioscience	Cat#12-4777-42; RRID:AB_1944444
CD4 (S3.5)	Invitrogen	Cat#MHCD0417; RRID:AB_10371766
CD28 (CD28.2)	BD Biosciences	Cat#561791; RRID:AB_10898345
CD57 (TB01)	eBioscience	Cat#25-0577-42; RRID:AB_2573354
IL-8 (E8N1)	BioLegend	Cat#511406; RRID:AB_893462
IL-17 (BL168)	BioLegend	Cat#512312; RRID:AB_961392
CD14 (MΦP9)	BD Biosciences	Cat#563079; RRID:AB_2737993
IL-2 (MQ1-17H12)	BD Biosciences	Cat#564165; RRID:AB_2738636
CD45RA (HI100)	BD Biosciences	Cat#563963; RRID:AB_2738514
IL-10 (JES3-9D7)	BD Biosciences	Cat#564050; RRID:AB_2738564
TNFa (Mab11)	BioLegend	Cat#502948; RRID:AB_2565858
IL-6 (MQ2-13A5)	BD Biosciences	Cat#561441; RRID:AB_10679121
CD69 (FN50)	BioLegend	Cat#310914; RRID:AB_314849
CD107a (H4A3)	BD Biosciences	Cat#562628; RRID:AB_2737686
IFN-g (B27)	BD Biosciences	Cat#557643; RRID:AB_396760

**Chemicals, peptides, and recombinant proteins**		

Enterotoxin Type B from *Staphylococcus aureus*	List Biological Laboratories	Cat#122
Phusion High-Fidelity PCR Master Mix with HF Buffer	New England Biolabs	Cat#M0531L

**Critical commercial assays**		

Quant-IT PicoGreen dsDNA Assay	Thermo Fisher	Cat#P7589
SequalPrepTM Normalization Plate Kit	Invitrogen	Cat#A1051001
Quick-DNA Fecal/Soil Microbe MiniPrep Kit	Zymo Research	Cat#D6010
Fixable Aqua Dead Cell Stain Kit, for 405 nm excitation	Thermo Fisher	Cat#L34957
eBioscience Foxp3/Transcription Factor Staining Buffer Set	Thermo Fisher	Cat#00-5523-00
BD Cytofix/Cytoperm Kit	BD Biosciences	Cat#554714; RRID: AB_2869008
BD GolgiPlug	BD Biosciences	Cat#555029; RRID: AB_2869014
BD GolgiStop	BD Biosciences	Cat#554724; RRID: AB_2869012
Rainbow Calibration Particles, 6th peak from the 8 peak set	Spherotech	Cat#RCP-30-5A-6
AgPath-ID™ One-Step RT-PCR Reagents	Applied Biosystems	Cat#4387424
Black Hole Quencher-1	Biosearch Technologies	3'-BHQ-1 CPG

**Deposited data**		

Annotated datasets for:-16S gene sequencing-flow cytometry results	dbGaP	phs001347

**Oligonucleotides**		

Forward primer V3-V4 rRNA sequencing (319F): 5'-ACT CCT ACG GGA GGC AGC AG-3'	Grier et al. (2018)	N/A
Reverse primer V3-V4 rRNA sequencing(806R): 5'-GGA CTA CHV GGG TWT CTA AT-3'	Grier et al. (2018)	N/A
Forward primer V1-V3 rRNA sequencing(8F): 5'-AGA GTT TGA TCC TGG CTC AG-3'	Grier et al. (2018)	N/A
Reverse primer V1-V3 rRNA sequencing(534R): 5'-ATT ACC GCG GCT GCT GG-3'	Grier et al. (2018)	N/A
Forward primer, UL55: 5'-TGG GCG AGG ACA ACG AA	Boeckh et al. (2004)	N/A
Reverse primer, UL55: 5'-TGA GGC TGG GAA GCT GAC AT	Boeckh et al. (2004)	N/A
Forward primer, UL123-exon4: 5'-TCC CGC TTA TCC TCR GGT ACA	Boeckh et al. (2004)	N/A
Reverse primer, UL123-exon4: 5'-TGA GCC TTT CGA GGA SAT GAA	Boeckh et al. (2004)	N/A

**Software and algorithms**		

Code and interim data	github (zenodo)	https://doi.org/10.5281/zenodo.5786917

### Resource availability

#### Lead contact

Further information and requests for resources and reagents should be directed to and will be fulfilled by the lead contact, Kristin Scheible (Kristin_Scheible@URMC.Rochester.edu).

#### Materials availability

This study did not generate new unique reagents.

### Experimental model and subject details

#### Study design and sample collection

All study procedures were approved by the University of Rochester Medical Center (URMC) Internal Review Board (IRB) (Protocol # RPRC00045470 & 37933) and all subjects' caregivers provided informed consent. The infants included in the study were enrolled within 7 days of life for the University of Rochester Respiratory Pathogens Research Center PRISM and were cared for in the URMC Golisano Children’s Hospital. Clinical data including nutrition, respiratory support, respiratory symptoms, medications, comorbidities, were entered into REDCap (Harris et al., 2009, 2019), then integrated with laboratory results using the URMC Bio Lab Informatics Server, a web-based data management system using the open source LabKey Server (Nelson et al., 2011). Blood was collected at birth, time of NICU discharge or 36-42 weeks PMA (whichever occurred first), and at 12 months of life. We collected 2729 gut (842 from NICU and 1887 post-discharge), and 2210 nasal (619 from NICU and 1591 post-discharge) usable microbiota samples longitudinally from 139 pre-term and 98 full-term infants and worked with the most extensive subset of these possible depending on the analysis in question (Table S2). From the PRISM study cohort, fecal (rectal) and nasal material was collected from pre-term infants (23 to 37 weeks gestational age at birth (GA)) weekly from the first week of life until hospital discharge, and then monthly through one year of gestationally corrected age. Rectal and nasal samples were collected from full-term infants at enrollment and monthly through one year. Additionally, rectal and nasal samples were collected from all infants whenever they exhibited symptoms of acute respiratory illness after discharge from the hospital. Symptoms of acute respiratory illness prompting sample collection were summarized by the primary caregiver using a symptom COAST (Childhood Origins of Asthma) score sheet (Busse et al., 2010). Parents were instructed to notify the study team if the infant had symptom score of three or greater. Demographic information on the cohort can be found in Table 1.

### Method details

#### Flow cytometry methods

Sample collection, isolation, storage, thawing, stimulation and staining for flow cytometry was performed as detailed previously (Scheible et al., 2012). In short, cord blood and peripheral blood mononuclear cells were isolated via Ficoll density gradient centrifugation, cryopreserved and stored in liquid nitrogen, and rapidly thawed and washed with pre-warmed RPMI-1640 supplemented with 10% FBS and 1x L-glutamine; thawing was done in ‘subject-balanced’ batches (equal mix of pre and fullterm subjects, each with three time points). An aliquot of each freshly thawed sample was stained with a T-cell phenotyping (Tphe) panel with the remainder of the sample rested overnight in an incubator, and stimulated with 2μg/mL of *Staphylococcus aureus*, Enterotoxin Type B (SEB); following an initial 2-hour stimulation, samples were blocked using GolgiPlug and GolgiStop (manufacturer’s recommended concentrations), further stimulated for 8 hours and then stained with a T-cell functional panel (ICS). Panel compositions are as shown in Table S5 and as listed in the key resources table.

Staining steps were performed using the following parameters: 96-well V bottom plates (∼2.5x10^6^ cells per well); 50 μL stain volumes; 200 μL wash volumes; 5-minute spins at 500 g; and 30-minute incubations (room temperature, in the dark). For both panels, plated samples were washed twice with PBS supplemented with 2% FBS (Stain Buffer), stained to assess viability (Fixable Aqua), washed once with Stain Buffer, and then surface stained. Following two washes with Stain Buffer, samples were fixed and permeabilized according to manufacturer’s protocol using the eBioscience FoxP3 Fix/Perm kit (Tphe panel) or the BD Cytofix/Cytoperm kit (ICS panel). Following two washes with the respective permeabilization buffer, samples were intracellular/intranuclear stained. Finally, samples were washed twice with permeabilization buffer and once with Stain Buffer.

Samples were acquired on a BD LSRII (core facility instrument QC-ed daily with BD CS&T beads); PMT voltages were normalized per run to pre-determined/optimized ‘Peak-6’ (Spherotech) median fluorescence values. All blood samples generating usable data were included in all analyses. Sufficient blood to perform T-cell phenotyping by flow cytometry at three pre-defined timepoints was collected from 55% of subjects at birth, 61% of subjects at NICU discharge, and 38% at 12 months. For training the PMA predictions models (described below), all microbiota samples were used. For all other analyses, microbiota samples from subjects that did not have any usable data from blood were excluded. Two staining panels, covering i) intracellular cytokine production (ICS) and ii) T-cell surface phenotyping (Tphe) were designed (Table S5). Complete immunophenotyping for all three timepoints was performed on 25% of subjects, and 63% of subjects had complete immunophenotyping for at least one timepoint.

#### Microbiota collection and sequencing

Microbiota sample collection and storage techniques, genomic DNA extraction and background control methods were as previously published (Grier et al., 2018). In brief, each sample was collected by inserting a sterile, normal saline moistened, flocked nylon swab beyond the sphincter into the rectum, for gut samples, or into the anterior nostril for nasal swabs, and twirling prior to removal. Each swab was then immediately placed into sterile buffered saline and stored at 4°C for no more than 4 h. Extraction of the fecal and nasal material from the swabs occurred daily in a sterile environment and were transferred immediately to  -80°C storage until DNA extraction. Genomic DNA was extracted from the nose, throat, and rectal samples using a modification of the Zymo Fecal/Soil Microbe Miniprep Kit and FastPrep mechanical lysis, and amplified with 16S rRNA gene primers as previously described (Grier et al., 2017, 2018). Briefly, amplification of 16S ribosomal RNA (rRNA) was performed using Phusion High-Fidelity polymerase (Thermo Scientific, Waltham, MA) and dual indexed primers specific to the V3-V4 hypervariable regions (319F: 5′ ACTCCTACGGGAGGCAGCAG 3′; 806R: 3′ ACTCCTACGGGAGGCAGCAG 5′). After pooling, amplicons were paired-end sequenced on an Illumina MiSeq (Illumina, San Diego, CA) in the University of Rochester Genomics Research Center. Positive controls for each sequencing run consisted of a 1:5 mixture of *Staphylococcus aureus*, *Lactococcus lactis*, *Porphyromonas gingivalis*, *Streptococcus mutans*, and *Escherichia coli* and negative controls consisted of sterile saline.

#### Cytomegalovirus detection

Subject saliva samples collected at 12 months of age were screened for cytomegalovirus (CMV) infection by Real-Time PCR using a double-primer assay targeting the UL55-UL123-exon 4 regions, according to a previously published protocol (Boeckh et al., 2004). Briefly, probes were labeled at the 5’ end with 6-carboxyfluorescein (FAM) and at the 3’ end with Black Hole Quencher^TM^1 (Biosearch Technologies, Inc., Novato, CA). Real-time assays were run on the Applied BiosystemsTM 7500 Real-Time PCR system (ThermoFisher Scientific) under the following conditions: 50°C for 10 min, 95°C for 10 min, followed by 45 cycles of 95°C for 20 s and 60°C for 1 min. Each 25 μl was prepared using AgPath-IDTM One-Step RT-PCR reagents (Applied Biosystems), to include 12.5 μl of 2x RT-PCR Buffer, 1 μl of 25x RT-PCR Enzyme mix, a 400 nM concentration of primers and 5 μl of sample extract. Sample quality was assessed using human RNaseP reference gene control (Boeckh et al., 2004). Positive control included known positive clinical isolate, primers and probes, and negative control was nuclease-free water.

### Quantification and statistical analysis

#### Flow cytometry gating and clustering

R-based packages and scripts were used for all post-acquisition processing and analysis of flow cytometry data. Reading of raw .fcs files, compensation, transformation, and subsetting/writing of .fcs files was performed using flowCore (Hahne et al., 2009). To minimize inter-run variation associated with the Tphe panel, the flowStats (Hahne et al., 2019) warpSet function was used to normalize arcsinh transformed channel data using a standard healthy donor adult PBMC control across batches as a reference. For analysis with the clustering algorithm FlowSOM, an iterative approach was used for both panels to first cluster on live, intact, lymphoid-sized CD4^+^ and CD8^+^ T-cell subsets (in the case of the ICS panel, including activated, CD69^+^ subsets); those subsets were then re-clustered to capture rare populations and optimally resolve phenotypic heterogeneity and associated function. Over-clustering followed by expert-guided merging was favored when defining the final number of T cell populations. FlowSOM clustering results used in downstream analysis were represented as proportion of the respective T-cell subset, per sample.

#### Microbiota identification

Raw data from the Illumina MiSeq was first converted into FASTQ format 2 × 312 paired-end sequence files using the bcl2fastq program (v1.8.4) provided by Illumina. Format conversion was performed without de-multiplexing, and the EAMMS algorithm was disabled. All other settings were default. Samples were multiplexed using a configuration described previously (Fadrosh et al., 2014). The *extract_barcodes*.py script from QIIME (v1.9.1) (Caporaso et al., 2010) was used to split read and barcode sequences into separate files suitable for import into QIIME 2 (v2018.11) (Bolyen et al., 2019) which was used to perform all subsequent read processing and characterization of sample composition. Reads were demultiplexed requiring exact barcode matches, and 16S gene primers were removed allowing 20% mismatches and requiring a matching window of at least 18 bases. Cleaning, joining, and denoising were performed using DADA2 (Callahan et al., 2016): reads were truncated (forward reads to 260 bps and reverse reads to 240 bps for rectal V3-V4 samples and forward reads to 275 bps and reverse reads to 260 bps for nasal V1-V3 samples), error profiles were learned with a sample of one million reads per sequencing run, and a maximum expected error of two was allowed. Taxonomic classification was performed with naïve Bayesian classifiers trained on target-region specific subsets of the August, 2013 release of GreenGenes (Desantis et al., 2006). Sequence variants that failed to classify to the phylum level or deeper were discarded. Sequencing variants observed fewer than ten times total, or in only one sample, were discarded. Rectal samples with fewer than 2250 reads and nasal samples with fewer than 1200 reads were discarded. Phylogenetic trees were constructed for each body site using MAFFT (Katoh and Standley, 2013) for sequence alignment and FastTree (Price et al., 2010) for tree construction. For the purposes of β-diversity analysis, rectal and nasal samples were rarefied to depths of 2250 and 1200 reads, respectively, and the Unweighted Unifrac (Lozupone et al., 2011) metric was applied.

#### Statistical analyses

##### Multivariate ANOVA

We used a sequence of multivariate ANOVA (MANOVA) models to estimate the amount of variance one set of variables could explain in another. We modeled T cell population relative abundances, gut, and nasal species-level relative abundances pairwise each as predictor and response matrices. DOL and PMA served only as predictors. Each pair of variables types was joined, with missing samples deleted case-wise. T cell populations and microbiome taxa with a variance of less than .0001 were removed in each comparison. The remaining variables were renormalized to sum to one, and transformed using the isometric log ratio, then modeled using a multivariate linear model with a matrix response. $R_{\text{adj}}^{2}$ was calculated as $1-MSE_{\text{full}}/MSE_{\text{reduced}}$ where the mean squared error (MSE) was the total sum of squared residuals in the response matrix, divided by the residual degrees of freedom, thus approximately unbiased for the residual variance. The PMA-adjusted model used PMA, and the set variables of interest as a predictor in the full model, retaining only PMA in the reduced model. Wilks’ lambda was used to test for association between response and predictor variables. The BIC was calculated as −2loglik(β)+nlogp, where n is the number of multivariate observations, p is the number of parameters in the model, and loglik(β) is the log-likelihood of the multivariate normal distribution with covariance derived from the variance-covariance matrix of the residual matrix.

##### CST and IST assembly

Microbial community state types (CSTs) were defined for each body site by fitting Dirichlet multinomial mixture (DMM) models (Holmes et al., 2012) using the R package DirichletMultinomial (v1.22.0) (Morgan, 2019; R Core Team, 2013), R version 3.5.0. Sample composition was represented using normalized counts of the most specific operational taxonomic units (OTUs) present in at least 5% of the samples from a given body site. Normalization was performed on a per sample basis by taking the relative abundance of each OTU and multiplying a constant, which we took as our QC cutoff on the minimum number of reads (2250 for rectal samples and 1200 for nasal samples). Resulting non-integer counts were rounded down. We also attempted modeling the un-normalized counts, but found that high-count libraries appeared to exert undue influence on the DMM model. For each body site, the DMM model was fit with one through twenty Dirichlet components and the optimal number of components was selected by minimizing the Laplace approximation of the negative-log model evidence. In this model, CSTs are synonymous with Dirichlet components, and each sample was assigned to the CST from which it had the highest posterior probability of be derived. This procedure was repeated with the immunological data in order to define immune state types (ISTs), using relative abundances of FlowSOM defined T cell populations in the place of OTUs. Relative abundances were computed within assays (TPHE and ICS) and major populations (CD4 and CD8) separately, and converted to counts by multiplying by 50,000 and rounding down. CD4 and CD8 counts were combined to fit the DMM for each assay.

##### CST and IST associations

To test for enrichment in pre- or full-term samples, we modeled the number of samples each subject had categorized into a CST/IST vs the total number of samples for the subject using logistic mixed models:

(#samplesinCST,# samplesnotinCST)∼gestationalageatbirth+confounders+(1|Subject),

where confounders were mode of delivery, maternal antibiotic use during pregnancy, number of months >50% feedings of breastmilk, any breastmilk before first discharge, infant antibiotics before discharge (days), and infant antibiotics after discharge (courses). In testing for IST associations with CMV, and other exposures, we modeled presence of the IST ever for the subject as a smooth function of the covariates of interest, and confounders: gestational age at birth, mode of delivery, race, and sex. In testing for associations with CMV and inflammation, we modeled the joint effect of maternal antibiotic use during pregnancy, clinical chorioamnionitis, membrane rupture >18 hours before birth and CMV. In three other separate models, we considered milk as an exposure (number of months >50% feedings of breastmilk, any breastmilk before first discharge), days of infant antibiotics before discharge, and courses infant antibiotics after discharge. For each variable of interest, we adjusted for multiple comparisons using the false discovery rate, and only reported findings significant at < 10% FDR.

##### Microbiota-T cell associations

Associations between microbiome development and the immune system were modeled using microbiome CST occurrence patterns as outcome variables and iterating through the relative abundances of each FlowSOM T cell population or observed IST at each time point as predictors. In symbols, we modeled

CST days∼immune_parameter+confounders+sampling_intensity,

using quasi-Poisson regression.

For each CST, each of these immunological parameters (T cell population relative abundances and IST, hereafter referred to as the immunological variables of interest [VOIs]) at each of the three time points when the immune system was sampled (birth, discharge, and one year) was assessed one-at-a-time. CST occurrence patterns were related to immunological VOIs by testing associations between every CST-VOI combination at the level of individual subjects, while controlling for confounders. In the most general model, these were: mode of delivery, gestational age at birth, perinatal breastmilk (yes/no), number of months after discharge more than 50% feedings were from breastmilk, number of days of antibiotics while in hospital, number of courses of antibiotics after discharge, and the log number of days observed (sampling_intensity) in each individual. Significance of the VOI was assessed with a Chisq-test, comparing the change in deviance when dropping the VOI from the model.

The response variable, the number of days a subject was assigned to a given CST was calculated by summing the interval lengths between CST change points. Intervals were calculated from midpoint to midpoint on the sampled days of life. At birth, subjects were placed in the first observed CST if the first sample occurred within 14 days of life, otherwise the first interval was excluded. Subjects were assumed to remain in their final observed CST for an interval equal to half the interval length between the penultimate and ultimate sample. Subjects with fewer than one sample taken per 30 NICU-days or fewer than six samples post discharge were excluded. We filtered immune VOI with fewer than ten observations, and CSTs present in fewer than 10% of the remaining observations. Numerical covariates were converted into z-scores, except gestational age (in weeks) which we modeled as gestational age −37 weeks. Multiple testing across all CSTs and VOIs was corrected for using the Benjamini-Hochberg method at 10% FDR.

##### Prediction of PMA

Two separate elastic net regression models (Friedman et al., 2010) were trained to predict (Bischl et al., 2000) the log2-transformed PMA with a) T cell immunological features and b) microbial OTU relative abundance. In (a) the four feature sets were CD4 ICS, CD8 ICS, CD4 Tphe and CD8 Tphe populations, while in (b) the two feature sets consisted of nasal and rectal species-level relative abundances from samples collected prior to DOL 450, filtered to remove taxa present in fewer than 3% of samples. A total of 433 samples from 185 subjects and 80 features were included in (a). Model (b) was trained on 3032 samples from 237 subjects and 218 features. Some samples had incomplete feature sets, e.g., if only the ICS panel was run then both the CD4 and CD8 Tphe sets were missing, or if only the nasal microbiome was sampled and the gut microbiome measures were missing. We treated this as a missing data problem, and imputed the values with their mean values among non-missing cases. Imputation was chained onto the elasticnet model (occurred only using the training data, in each fold) for the purposes of tuning and validation. Within each feature set, we used the relative proportions, transformed into z-scores.

##### Cross validation for tuning and prediction

We tuned the model and estimated its performance using cross-validation by holding out a subject’s entire longitudinal record. We tuned the elastic net alpha in [0, 1] and lambda in [.001, .5] parameters by randomly selecting 50 combinations of (alpha, lambda) and evaluating the test mean-square error (MSE) via 5-fold cross-validation. After finding a minimizing pair of (alpha, lambda), the model was refit with 10-fold cross-validation. For each subject i, this provides two sequences of fitted values, representing the log2-transformed PMA prediction. For instance, for the microbiome, we have

$$\hat{Y_{ij}}=f^{-i}\left(x_{ij}\right),\;j=1,\ldots ,n_{i},$$

where *x*_*ij*_ represent microbial feature vectors, *n*_*i*_ indexes the number of longitudinal samples for subject *i*, and *f*^*−i*^ represent the elastic net model trained excluding subject i. For the T cell immunome, the analogous model is fit. The back-transformed values $2^{\hat{Y_{ij}}}$ were used to calculate each model’s *R*^*2*^.

##### Immunological and microbial developmental indices

The longitudinal sequence of cross-validated fitted values $\hat{Y_{ij}}$ were compared to the true PMA for each subject using a linear mixed model. We fit the model

$$\hat{Y}-{\log\;}_{2}\left(37\right)∼{\log\;}_{2}\left(PMA/37\right)+\left(1+{\log\;}_{2}\left(PMA/37\right)|\text{Subject}\right)$$

thus $\hat{Y_{ij}}=\alpha_{i}+\beta_{i}\times PMA_{ij}+\epsilon_{ij}$ and calculated the best linear unbiased predictor of each subject’s 37-week intercept $\alpha_{i}$, slope $\beta_{i}$ and their conditional standard errors se$\left(\alpha_{i}\right)$, se$\left(\beta_{i}\right)$. These are transformed into a quantity similar to a z-score by subtracting the median of $\alpha_{i},\;\beta_{i}$ over subjects i, and dividing by its conditional standard error se$\left(\alpha_{i}\right)$ or se$\left(\beta_{i}\right)$.

##### Prediction of PRD

We used random forest classification models to predict PRD using two feature sets: clinical and developmental index. The clinical features were race, maternal education, the baby’s sex, gestational age, weight and season at birth, and oxygen supplementation integrated over the first 14 days of life. The developmental index features were the z-scores of the microbiome and T-immune slopes and intercepts. The random forest hyperparameters mtry, ntree and nodesize were tuned separately for each feature set with random search using 5-fold cross-validation. After the optimal parameters were found for each feature set, a second round of 20-fold cross validation was used to evaluate the area under the ROC curve (AUC). The fitted values from the random forest regression were calculated using the function generatePartialDependenceData.

### Additional resources

This prospective, observational cohort study is registered in ClinicalTrials.gov (NCT01789268) under the official title “Impact of Respiratory Virus Infections and Bacterial Microbiome Shifts on Lymphocyte and Respiratory Function in Infants Born Prematurely or Full Term.” Dr. Gloria Pryhuber, co-author, is the trial’s responsible party, and other study details can be found at https://clinicaltrials.gov/ct2/show/NCT01789268.

## Data Availability

• Flow cytometry (.ics) and 16S sequencing data have been deposited at dbGaP accession dbGap: phs001347 and are available to other researchers, subject to dbGaP procedures as of the date of publication. • Data and code supporting this analysis has been deposited with Zenodo, under doi https://doi.org/10.5281/zenodo.5786917 and are publicly available as of the date of publication. • Any additional information required to reanalyze the data reported in this paper is available from the lead contact upon request.
