# Supplementary material for: Aberrant newborn T cell and microbiota developmental trajectories predict respiratory compromise during infancy
Source: iScience. 2022 Mar 1;25(4):104007. doi: 10.1016/j.isci.2022.104007 (PMC8931366; doi:10.1016/j.isci.2022.104007)
Supplement: Document S1. Figures S1–S6 and Tables S1–S5 [file mmc1.pdf]

iScience, Volume *25*

Supplemental information

Aberrant newborn T cell and microbiota developmental trajectories predict respiratory compromise during infancy

Andrew McDavid, Nathan Laniewski, Alex Grier, Ann L. Gill, Haeja A. Kessler, Heidie Huyck, Elizabeth Carbonell, Jeanne Holden-Wiltse, Sanjukta Bandyopadhyay, Jennifer Carnahan, Andrew M. Dylag, David J. Topham, Ann R. Falsey, Mary T. Caserta, Gloria S. Pryhuber, Steven R. Gill, and Kristin M. Scheible

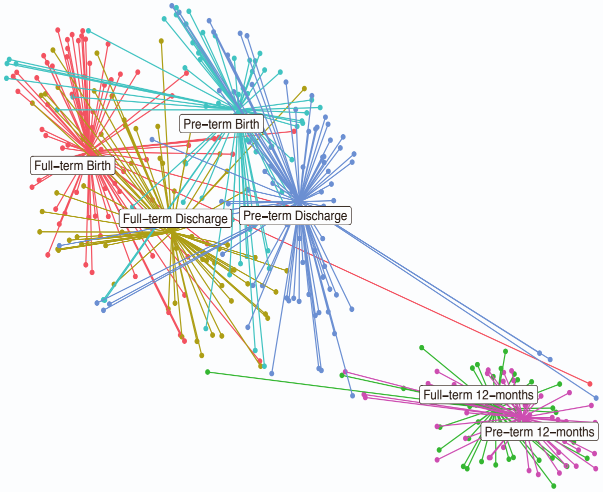

Supplementary figure 1, related to figure 2. UMAP of representing the relative abundances of 80 T cell populations. Each point represents a sample, and is colored according to the timepoint of sampling and cohort.

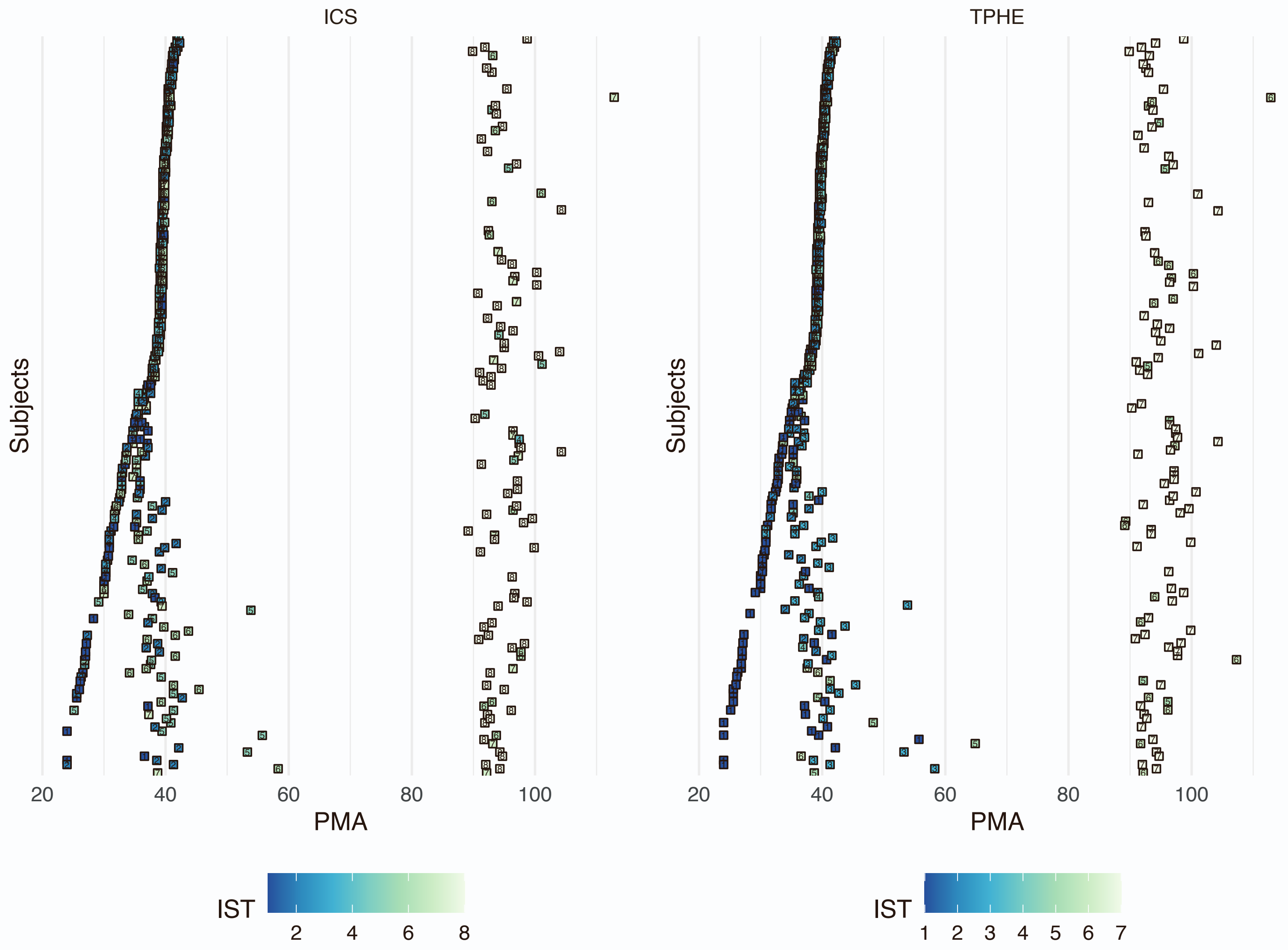

Supplementary figure 2, related to figure 3. Time points, as measured in post-menstrual age (PMA) when subjects (rows) were sampled for T cell ICS, T cell TPHE. The immune state type assigned to that sample is indicated by the hue.

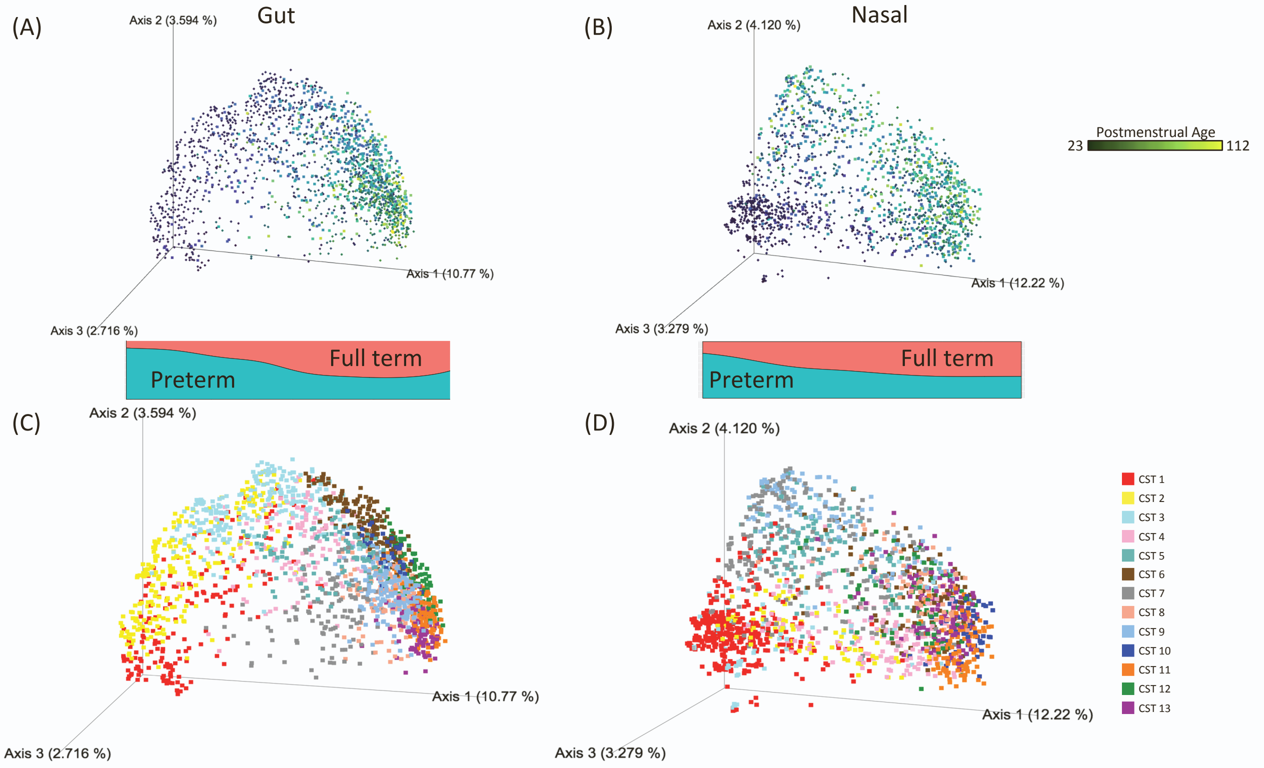

Supplementary figure 3, related to figure 4. Nasal and gut microbiota shifts in the first year of life among preterm and full term infants track with postmenstrual age. Microbiota community profiling was performed on rectal (A, C) and nasal (B, D) samples obtained from 159 infants during regular surveillance and acute respiratory illness. (A-D) Principal coordinate analysis (PCOA) plots using Unweighted Unifrac distances summarize overall variation and structure. (A-B) Points were colored by postmenstrual age (PMA) at the time the sample was obtained. Colored bands at the base of PCOA plots show the proportion of samples along each point of axis 1 that are from either preterm (teal) or full term (salmon) subjects. (C and D) Microbiota community state types (CST) were defined for each body site, with samples in the PCOA colored according to the CST they represent. CSTs are ordered according to average PMA of occurrence.

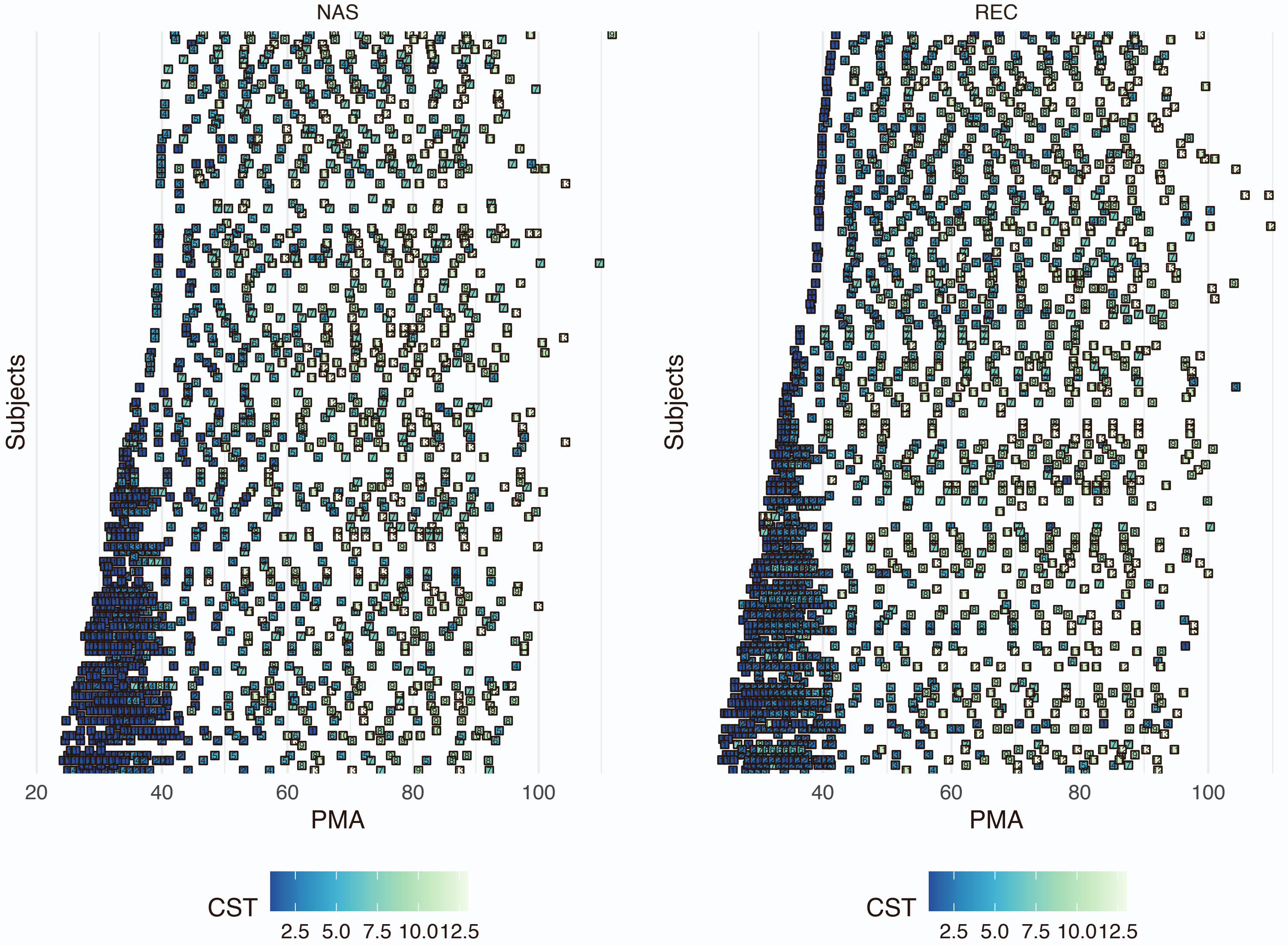

Supplementary figure 4, related to figure 4. Time points, as measured in post-menstrual age (PMA) when subjects (rows) were sampled for nasal or rectal microbiome profiling. The community state type assigned to that sample is indicated by the hue.

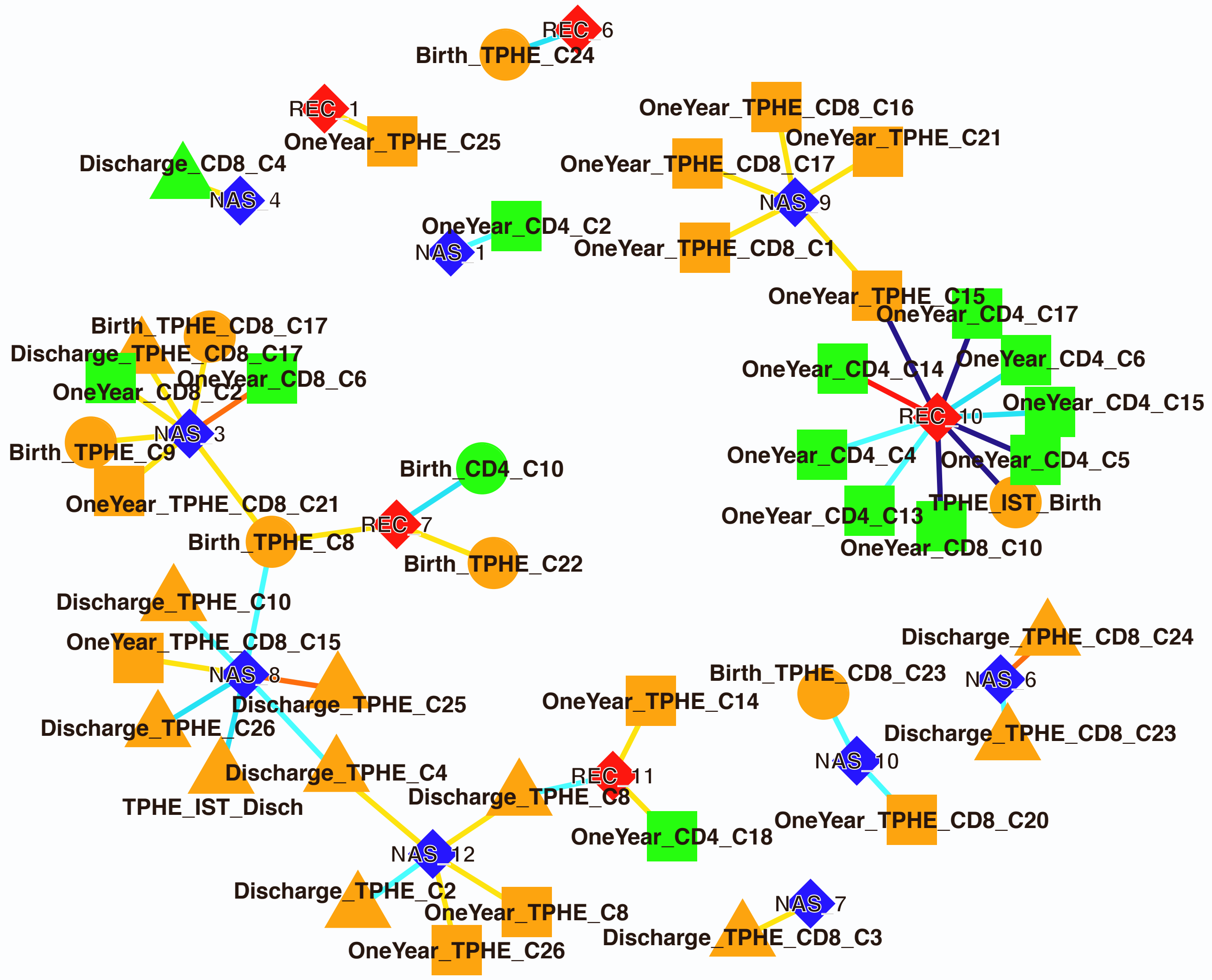

Supplementary figure 5, related to figure 5. Hub-like associations between duration of microbiota exposure and T cell phenotype in the first year. Associations between the number of days spent in microbiome states and T cell state estimated using Poisson regression, adjusting for gestational age at birth, breastmilk exposure, days of antibiotic exposure, mode of delivery and number of days covered by microbiome sampling. Edges are colored blue for inverse associations (increased abundance in T cell state decreases duration of microbiome state) and yellow-red for positive associations. Edges are shown for associations significant at 10% FDR over 6318 comparisons.

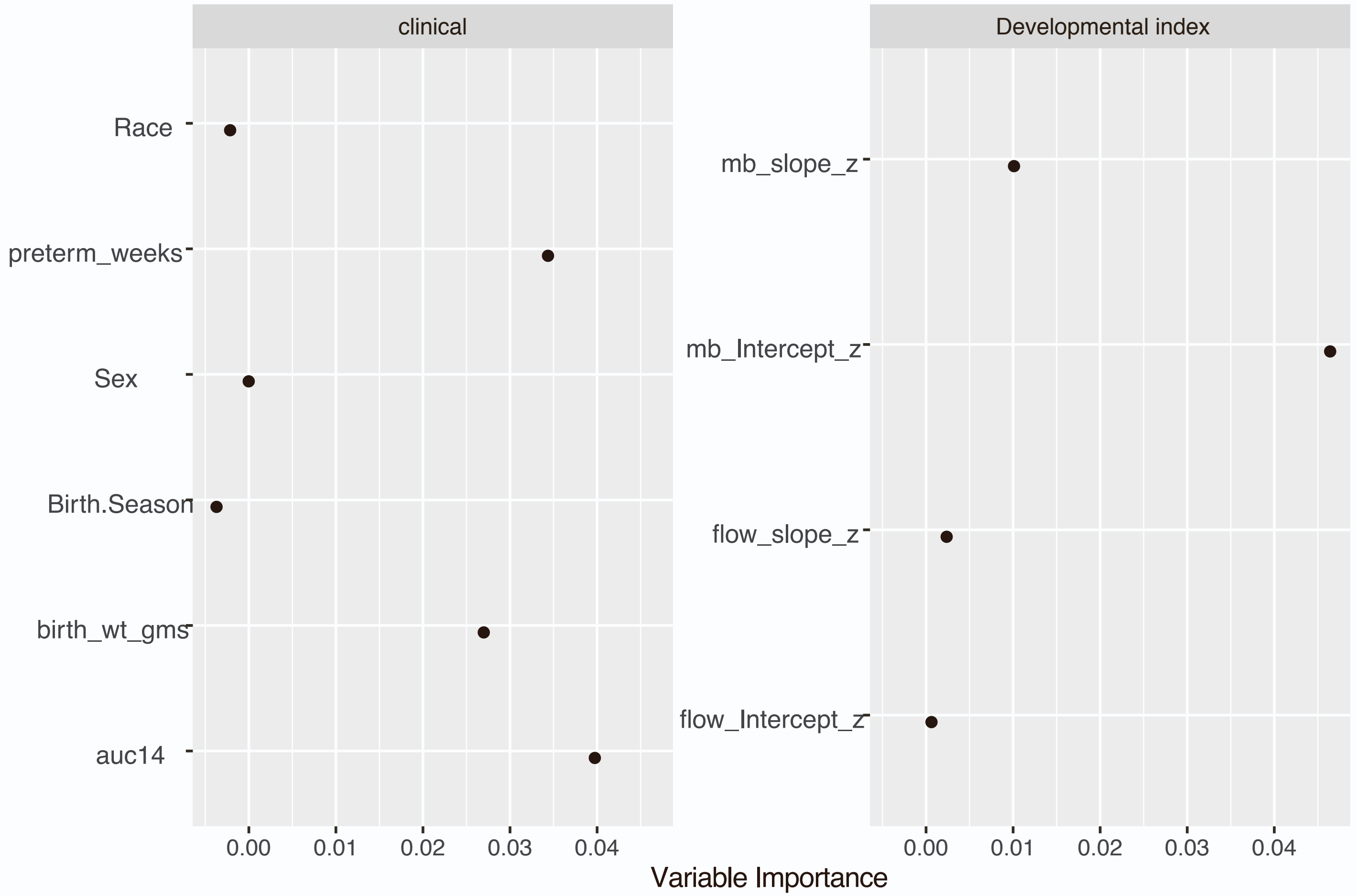

Supplementary fig 6, related to figure 6. Random forest variable importance plots for clinical and developmental index models. Larger values represent greater decreases in the Gini purity coefficient. Importance was calculated using the default method in R package randomForestSRC version 2.7.0.

TPHE	subjects	samples
3 timepoints	78	234
2 timepoints	82	164
1 timepoint	16	16
total	**176**	**414**

TPHE	samples	pre-term	full-term
birth	147	65	82
discharge	163	87	76
12-month	104	58	46
total	**414**	*210*	*204*

ICS	subjects	samples
3 timepoints	69	207
2 timepoints	89	178
1 timepoint	19	19
total	**177**	**404**

ICS	samples	pre-term	full-term
birth	147	64	83
discharge	155	84	71
12-month	102	55	47
total	**404**	*203*	*201*

TPHE_ICS	subjects	samples
3 timepoints	67	201
2 timepoints	83	166
1 timepoint	18	18
total	**168**	**385**

TPHE_ICS	samples	pre-term	full-term
birth	141	63	78
discharge	150	81	69
12-month	94	51	43
total	**385**	*195*	*190*

Supplementary Table 1, related to figures 1-3, 5-6. Subject numbers for immunophenotyping,

Panels Measured				
Number of timepoints	Cohort	ICS only	TPHE only	ICS and TPHE
1	Full term	0	1	6
	Pre term	1	2	10
2	Full term	5	0	36
	Pre term	2	4	47
3	Full term	0	2	37
	Pre term	1	1	30

Supplementary Table 2, related to figures 1-3, 5-6. Number of timepoints measured per subject by T cell flow cytometry panel by term.

Figure Description	Site	Samples	Subjects
**Microbiome CGA & CST PCoA plots**	Both NAS	1748	149
	Both REC	1899	143
**Microbiome CST Occurrence Over CGA**	NAS	1748	149
	REC	1899	143
**Microbiome Composition Heatmaps**	NAS	1748	149
	REC	1899	143
**Immuno IST Composition Heatmaps**	TPHE	414	176
	ICS	404	177
**Immuno IST Occurrence Over CGA**	TPHE	414	176
	ICS	404	177
**Immuno IST Avg. Occurrence GAB/CGA**	TPHE	414	176
	ICS	404	177
**NAS 8 Occurrence vs TPHE ISTs**	Birth	68	68
	Discharge	95	95
**CST-Immuno Association Networks**	NAS	1589	109
	REC	1697	117

Supplementary Table 3, related to figures 1, 4-6. Subject numbers microbiome and combined analyses.

Predictors	Response	BIC	Adjusted R2	Total R2	log10(p) vs worst model
DOL + Term	T cell	10,900	0.166	0.184	0
DOL + Term	NAS	212,436	0.055	0.075	0
DOL + Term	REC	599,240	0.028	0.036	0
PMA + Term	T cell	10,786	0.179	0.197	-50
PMA + Term	NAS	212,085	0.066	0.085	-153
PMA + Term	REC	599,023	0.031	0.038	-94
DOL + PMA + Term	T cell	10,606	0.017	0.211	-128
DOL + PMA + Term	NAS	211,896	0.007	0.091	-235
DOL + PMA + Term	REC	598,654	0.004	0.042	-255

Supplementary Table 4, related to figure 1. Model statistics for multivariate ANOVA. T cell population relative abundances, gut, and nasal species-level relative abundances were modeled as response variables, using indicated combinations of predictors. The BIC was calculated as –2*log-likelihood + p * log(n), where p is the number of predictors and n is the number of observations. The Adjusted R2 was calculated as *1*-MSEfull/MSEreduced, where MSEreduced is the mean square error in a model that excluded the first indicated predictor in the Predictors column. The total R2 uses the intercept-only in the reduced model. log10(p) vs worst model was calculated as log10(e) * [BIC – log(sum(exp(BIC))], the sum being over all combinations of models for that Response.

Cytometer: BDLSRII (URMC FlowCore - Animal)									
Tphe Functional Panel (RPRC 12-0012)									
Laser	Long Pass	Band Pass	PMT	Detector	Marker	Color	Clone	Company	Catalog #
488	505	515/20	BB	B515	**CD122**	BB 515	Mik-β	BD Biosciences	564688
488	685	710/50	BA	B710	**Perforin**	PerCP-Cy5.5	dG9	Biolegend	308114
407		450/50	VH	V450	**Granzyme B**	BV 421	GB11	BD Biosciences	563389
407	535	550/40	VG	V550	**Live/Dead**	Aqua		Life Technologies	L34957
407	570	585/42	VE	V585	**CD3**	BV 570	UCHT1	Biolegend	300436
407	595	605/40	VD	V605	**CD31**	BV 605	WM59	BD Biosciences	562855
407	630	660/40	VC	V660	**CD127**	BV 650	HIL-7R-M21	BD Biosciences	563225
407	670	705/70	VB	V705	**CD45RO**	BV 711	UCHL1	BD Biosciences	563722
407	740	780/60	VA	V780	**CD8a**	BV 785	RPA-T8	Biolegend	301045
633		660/20	RC	R660	**KLRG1**	APC	13F12F2	eBioscience	17-9488-42
633	685	710/50	RB	R710	**CD185 (CXCR**	APC-R700	RF8B2	BD Biosciences	565191
633	740	780/60	RA	R780	**CD197 (CCR7**	APC-Cy7	G043H7	Biolegend	353212
532		575/24	GE	G575	**Foxp3**	PE	236A/E7	eBioscience	12-4777-42
532	600	610/20	GD	G610	**CD4**	PE-TR	S3.5	Invitrogen	MHCD0417
532	640	660/40	GC	G660	**CD28**	PE-Cy5	CD28.2	BD Biosciences	561791
532	740	780/40	GA	G780	**CD57**	PE-Cy7	TB01	eBioscience	25-0577-42

ICS Functional Panel (RPRC 12-0012)									
Cytometer: BDLSRII (URMC FlowCore - Animal)									
Laser	Long Pass	Band Pass	PMT	Detector	Marker	Color	Clone	Company	Catalog #
488	505	515/20	BB	B515	**IL-8**	FITC	E8N1	BioLegend	511406
407		450/50	VH	V450	**IL-17**	Pacific Blue	BL168	BioLegend	512312
407	535	550/40	VG	V550	**Live/Dead**	Aqua	polyclonal	Life Technologies	L34957
407					**CD14**	BV510	MϕP9	BD Biosciences	563079
407	570	585/42	VE	V585	**CD8a**	BV570	RPA-T8	BioLegend	301037
407	595	605/40	VD	V605	**IL-2**	BV605	MQ1-17H12	BD Biosciences	564165
407	630	660/40	VC	V660	**CD45RA**	BV650	HI100	BD Biosciences	563963
407	670	705/70	VB	V705	**IL-10**	BV711	JES3-9D7	BD Biosciences	564050
407	740	780/60	VA	V780	**TNFa**	BV785	MAb11	BioLegend	502948
633		660/20	RC	R660	**IL-6**	APC	MQ2-13A5	BD Biosciences	561441
633	685	710/50	RB	R710	**CD3**	AF700	UCHT1	BD Biosciences	557943
633	740	780/60	RA	R780	**CD69**	APC-Cy7	FN50	BioLegend	310914
532		575/24	GE	G575	**IL-4**	PE	MP4-25D2		
532	600	610/20	GD	G610	**CD107a**	PE-CF594	H4A3	BD Biosciences	562628
532	690	710/50	GB	G710	**CD4**	PE-Cy5.5	S3.5	ThermoFischer	MHCD0418
532	740	780/40	GA	G780	**IFN-g**	PE-Cy7	B27	BD Biosciences	557643

Supplementary Table 5, related to figures 1-3, 5-6. Flow Cytometry Panels.
